# Joint SASMA and Bricscess Congress: Cape Town - South Africa, Thursday 10 – Sunday 13 October 2019

**Published:** 2019-01-01

## Background

The sit-to-stand (SiSt) and stand-to-sit (StSi) movements are repetitive daily activities which include loading of the hip, knee and ankle joints on each occasion. It is hypothesized that in amputees, compensation due to asymmetrical movements, can lead to joint overloading. This study aimed to describe the muscle activation levels and symmetry of vertical ground reaction force (vGRF) during the SiSt and StSi movements in unilateral transtibial amputees (UTTA).

## Method

A total number of 25 participants were recruited for the studying, consisting of 12 UTTA and 13 controls. Participants made use of their own prosthesis that they were familiar and comfortable with. Surface electromyography (EMG) and vGRF were recorded during ten continuous sit-to-stand-to-sit repetitions.

## Results

Muscle activation was significantly greater for UTTA lumbar erector spinae muscles (15.98±5.93%, p<0.05) during the SiSt and tibialis anterior (17.14±12.09%, p<0.05) during SiSt and StSi in comparison to the controls while affected side vastus lateralis activation was significantly lower (3.09±2.46%, p<0.05) than the unaffected and control sides. Significantly higher vGRF was observed on the unaffected side (69.90±5.77 N.kg^−1^) of the UTTA compared to the affected (52.29±5.45 N.kg^−1^) and control sides (p<0.05).

## Conclusions

Lower muscle activation of the vastus lateralis and lower vGRF on the affected side could be an indication of poor knee control or the inability of the UTTA to load the prosthesis during the SiSt and StSi. This indicates the possible presence of a combination of muscle weakness and lack of confidence in the use of the prosthesis. The asymmetrical movement pattern may place the amputee at increased risk for joint degeneration (osteoarthritis) over time. It would be suggested that specific rehabilitation once an amputee receives their prosthesis be followed to help reduce this risk.
