# Supplementary material for: Joint SASMA and Bricscess Congress: Cape Town - South Africa, Thursday 10 – Sunday 13 October 2019
Source: S Afr J Sports Med. 2019 Jan 1;31(1):v31i1a7466. (PMC9924525)
Supplement: Related texts

# Abstract: O02: Players’ experiences of the immediate management of rugby-related acute spinal cord injury in South Africa

**Published:** 2019-01-01

**Authors:** Marelise Badenhorst, Evert Verhagen, Mike Lambert, Willem van Mechelen, James Brown

**Affiliations:** 1 Division of Exercise Science and Sports Medicine, Department of Human Biology, University of Cape Town, Cape Town, South Africa; 2 Amsterdam Collaboration on Health and Safety in Sports, Department of Public and Occupational Health and Amsterdam Public Health research institute, Amsterdam UMC, Vrije University Amsterdam, The Netherlands; 3 Institute of Sport and Exercise Medicine (ISEM), Division of Orthopaedic Surgery, Department of Surgical Sciences, Faculty of Medicine and Health Sciences, Stellenbosch University; 4 International Olympic Committee (IOC) Research Centre, South Africa

## Introduction

The optimal management of rugby-related spinal cord injuries (SCIs) is challenging developing country with socio-economic and healthcare inequities and a detailed understanding of the different barriers and facilitators to optimal care is needed. The aim of this study was to describe the perception of players, regarding factors related to the optimal immediate management of a catastrophic injury, in the periods before and after the implementation of the national rugby safety programme, BokSmart.

## Methods

The entire known population (n=113) of players who had sustained a rugby-related SCI in South Africa in the period of 1968–2015, as recorded in the BokSmart/Chris Burger Petro Jackson Players Fund database (CBPJPF), formed the population of the study. Ninety players agreed to participate and were included in the broader study. From these 90 semi-structured interviews, stratified purposive sampling was used to select interviews from players from a variety of socio-economic status and geographical areas for inclusion in this study. The final sample consisted of forty-eight (n=48) participants.

## Results

Prominent barriers in the period prior to the implementation of BokSmart, such as inadequate equipment and the quality of first aid care appeared to have improved in the post-BokSmart period. However, the most frequently reported barriers, in the periods before and after the BokSmart, were transportation delays after injury and admission to appropriate medical facilities. Barriers were more prevalent in rural and lower socio-economic areas.

## Conclusion

These barriers can be improved by applying an appropriate action plan and determining the correct lines of communication to emergency medical services, all of which are described within the mandatory BokSmart course. The current findings should assist BokSmart to focus their resources on strategies that ensure the dissemination of knowledge into practices on field. These findings may help shape education, awareness and future policy around the immediate management of rugby-related SCIs.

# Abstract: O03: Mental health profiles of Para athletes in South Africa

**Published:** 2019-01-01

**Authors:** Marelise Badenhorst, Phoebe Runciman, James Brown, Lara Grobler, Wayne Derman

**Affiliations:** 1 Institute of Sport and Exercise Medicine (ISEM), Division of Orthopaedic Surgery, Department of Surgical Sciences, Faculty of Medicine and Health Sciences, Stellenbosch University, Cape Town, South Africa; 2 International Olympic Committee (IOC) Research Centre, South Africa; 3 Department of Sport Science, Faculty of Education, Stellenbosch University, Cape Town, South Africa

## Introduction

A comprehensive understanding of Para athlete health should include aspects such as mental health. However, there is a paucity of research investigating the mental health profiles of athletes with impairments in South Africa, as well as globally.

## Methods

Data were collected during the 2019 National Championships for athletes with impairments, which included athletes from provincial to international levels. Mental health was measured with the Trait component of the State/Trait Anxiety Inventory (STAI) and the Kessler Psychological Distress Scale (K-10). In both scales, lower scores indicate better mental health. On completion of the questionnaires, all athletes were given information of mental health support services. Between-group differences were analysed using the Mann-Whitney U test or one-way ANOVA.

## Results

A total of 124 athletes, with a mean age of 26.7 (±9.2) years, were included in the study. The mean score for the STAI was 39.2 (±9.3) units. A quarter (25.4%) of all athletes scored 45 and above, in line with scores of patients with a psychological / psychiatric diagnosis. There was a significant difference in STAI scores between females (35.9±8.7) and males (40.3 ±9.3; *p*=0.02), and between married (32.6±8.9) and single (40.2±8.9; *p*=0.002) athletes. The mean score of the K-10 questionnaire was 20.4 (±6.4) units, with no significant difference between genders. K-10 scores were significantly higher in single than in married athletes (*U* =415.5; *p* =0.002). Using a cut off score of ≥28 and ≥16 on the K-10, 12.2 % and 76.4 % of athletes had symptoms of distress, respectively. Neither scale was associated with level of competition, sport code or impairment type. The K-10 and STAI scores were highly correlated (r=0.64, p<0.001).

## Conclusion

The study found high rates of psychological distress among athletes with impairments. These findings demonstrate a need to understand and identify possible mechanisms affecting mental health in this population.

# Abstract: O04: Determinants of physical activity participation in Ethiopian people with disabilities

**Published:** 2019-01-01

**Authors:** Getachew Kinati Basha, Johan van Heerden

**Affiliations:** 1 University of Wollega, Ethiopia; 2 Department of Biokinetics, Exercise and Leisure Science, University of KwaZulu-Natal, South Africa

## Introduction

Encouraging physical activity participation is a public health imperative for all individuals but more so for those with a disability. Hence, it is important that stakeholders have knowledge of potential facilitators and barriers for physical activity participation, in specific population settings. The purpose of this study was to determine the key facilitators and barriers and to physical activity participation and their-related factors among people with vision and limb disabilities in Ethiopia.

## Methods

A descriptive questionnaire survey was done among individuals (n=334) with visual and limb impairment, aged between 15 and 50 years age, of both genders living in urban and suburban areas of Ethiopia. Multiple linear regression modelling was performed to identify key facilitators and barriers to physical activity and a Pearson chi-square test was subsequently done to establish associations between related demographic variables and the individual items of identified facilitators and barriers.

## Results

Time constraint and engagement was identified as a significant (p≤ 0.05) key barrier and facilitator, respectively for physical activity participation. Not being able to find the time to be physically active was associated with the female gender (38.1%; p≤0.05), the age category of 31–40 years (52.6%; p≤0.05), a diploma qualification (52.6%; p≤0.05) and aetiology of disability (50.0%; p≤0.05). Engagement factors were weight-loss associated with females (71.7%; p≤0.05), while weight maintenance, wanting to be physically fit and enjoying the challenge was associated (p≤0.001) with all age groups and levels of education. Weight maintenance was also associated with both disability type (p≤0.05) and cause of disability (p≤0.05), while the latter was likewise associated (p≤0.05) with enjoying the challenge. The modelling process showed that negating “time constraint” as a barrier to increasing physical activity participation would be more effective than facilitating “engagement” as a motivator for participation.

## Conclusions

Ethiopian stakeholders responsible for an action plan informing an awareness campaign to increase physical activity among people with limb and vision disability should primarily focus on educating individuals and employers to manage and decrease time constraints as a major barrier to physical activity participation

# Abstract: O05: Prevalence of lifestyle-related risk factors for non-communicable diseases in 1**^st^** year university medical students: preliminary data from the HELP-Health study

**Published:** 2019-01-01

**Authors:** Jill Borresen, Martin Schwellnus, Martin Bac, Debashis Basu, Gerda Gericke, Amanda Talma, Paola Wood, Kim Nolte, Tanya Camacho, Marianne Schwellnus

**Affiliations:** 1 Sport, Exercise Medicine and Lifestyle Institute (SEMLI), University of Pretoria, South Africa; 2 Department of Family Medicine, University of Pretoria, South Africa; 3 School of Health Systems and Public Health, University of Pretoria, South Africa; 4 Department of Human Nutrition, University of Pretoria, South Africa; 5 Division of Biokinetics and Sport Science, University of Pretoria, South Africa; 6 School of Medicine, University of Pretoria, South Africa

## Introduction

With the prevalence of non-communicable disease (NCDs) increasing, the majority of patients seen by healthcare professionals will be those with NCDs and associated risk factors. The health behaviours of healthcare professionals influence their counselling practices, their credibility in delivering lifestyle-related messages, and their success in guiding behaviours in patients; yet little is published on healthcare professionals and students. The WHO identifies physical inactivity, unhealthy diet, harmful use of alcohol and tobacco use as key behaviours to address. This study explores the prevalence of these risk factors in 1st year medical students.

## Methods

This is a cross-sectional, observational study in 284 consenting 1^st^ year MBChB and Bachelor of Clinical Medicine Practice (BCMP) students at the University of Pretoria (96 male, 188 female, mean age 19.9±2.7 years). The Rapid Eating Assessment for Patients (REAP), Frequency Intensity Time (FIT) Index of Kasari, a subset of questions from Global Adult Tobacco Survey (GATS), and the Alcohol Use Disorders Identification Test (AUDIT) were administered using Smartabase (Fusion Sport PtyLtd). Outcome variables included prevalence (%) of low physical activity, low fruit and vegetable intake, smoking, and excessive alcohol consumption. Chi square statistical test was performed to ascertain differences between male and female participants

## Results

66% reported engaging in low levels of habitual physical activity (males 61%, females 68%; not significantly different (NS)); 34% usually/often eat less than 2–3 fruits (males 40%, females 31%; NS) and 31% usually/often eat less than 3–4 vegetables a day (males 28%, females 32%; NS). 8% report smoking (males 13%, females 6%; p=0.045) and 12% reflect harmful alcohol use (males 20%, females 8%; p=0.006).

## Conclusions

These are preliminary data aimed at identifying lifestyle-related risk factors that should be addressed; thereby contributing to developing and implementing the HEalthy Lifestyle Programme for HEALTHcare professionals (HELP-HEALTH).

# Abstract: O06: Hamstring/quadriceps ratio and hip abduction strength imbalances in amateur rugby union players with or without previous injuries

**Published:** 2019-01-01

**Authors:** Micaela Brown, Lyle Kearns, Andre Pienaar, Rowena Naidoo

**Affiliations:** Discipline of Biokinetics, Exercise and Leisure Sciences, University of Kwa-Zulu Natal, Westville campus, South Africa

## Introduction

Hamstring/Quadriceps ratio (H/Q) and hip abduction strength are important for performance and injury prevention in amateur rugby players. The strength relationship and balance of co-activation between the flexor, extensor and abductor muscles are important in articular stabilisation, efficacy of movement and the prevention of injuries. This study aimed to measure the H/Q strength ratios and its relationship to hip abduction strength of the dominant and non-dominant leg; as well as to determine if there was a relationship between strength imbalances and previous lower limb injuries.

## Methods

This descriptive, correlational design study, comprised Sharks Academy rugby players, in KwaZulu-Natal. Fifty-nine players between the ages of 18 to 23 years, participated in this study. All participants completed an injury prevalence questionnaire and anthropometric (height and weight) measurements were taken. Knee flexion, knee extension and hip abduction were measured using the Pressure Air Feedback (PABR) system. Descriptive (means and standard deviations) and inferential (t-tests and Pearson correlation) statistics were used. (p≤0.05)

## Results

A positive correlation between H/Q ratio and hip abduction strength was found. No significant strength differences between dominant and non-dominant legs, as well as no correlation between the strength ratios and previous injuries were found. The players H/Q ratio was sufficient to meet the minimal standards, however hip abduction strength was weak.

## Conclusions

Although the history of lower limb injuries did not correlate with current strength ratios, the data recorded identified the player’s strengths and weaknesses. This data is useful to improve lower limb strength monitoring. The findings highlight the importance to monitor lower limb strength and to help improve conditioning and rehabilitation programmes for the amateur rugby players.

# Abstract: O07: The silver lining of the high rate of concussions in Stellenbosch koshuis rugby competition

**Published:** 2019-01-01

**Authors:** James Craig Brown, Lindsay Toyah Starling, Keith Stokes, Pierre Viviers, Esme Jordaan, Sean Surmon, Elton Wayne Derman

**Affiliations:** 1 Institute of Sport and Exercise Medicine, Division of Orthopaedics, Department of Surgical Sciences, Stellenbosch University, Cape Town, South Africa; 2 IOC Research Centre, South Africa; 3 Department for Health, University of Bath, Bath, United Kingdom; 4 Rugby Football Union, Twickenham, United Kingdom; 5 Biostatistics Unit, Medical Research Council, Cape Town, South Africa; 6 Statistics and Population Studies Department, University of the Western Cape, Cape Town, South Africa; 7 Maties Sport, Stellenbosch University, Stellenbosch, South Africa

## Introduction

Collision sports, such as Rugby Union (‘Rugby’) have a particularly high risk of injury. Despite non-professional Rugby players comprising the majority of the world’s playing population, there is relatively little research in this population. Stellenbosch Rugby Football Club (‘Maties’), the official rugby club of Stellenbosch University, represents one of the world’s largest non-professional Rugby clubs, making this an ideal cohort for community level injury surveillance. The aim of this study was to describe the incidence and events associated with concussion in this cohort.

## Methods

Baseline demographics were obtained on the 807 male student Rugby non-professional players who registered for the ten-week long 2018 season, which comprised 101 matches and 2 915 of exposure hours. All match-related injuries were captured by the medical staff of Stellenbosch Campus Health Service on an electronic form developed from the consensus statement for injury recording in Rugby.

## Results

The average age, height and weight of this cohort was 20±2 years, 182±7 cm and 88±14 kg, respectively. Overall, there were 89 time-loss injuries, which equated to an injury rate of 31 per 1000 match hours (95% confidence intervals [CIs]: 24–37), or about one injury per match. The most common injury diagnosis was “concussion” (n=27 out of 90 injuries, 30%), at a rate of 9 per 1000 match hours (95% CIs: 6–12). The three most common mechanisms of concussion in the present study were performing a tackle (33%), accidental collision (30%) and being tackled (11%).

## Conclusion

Concussion was the most common injury in this population, at a rate that was six times higher than a comparable cohort in the UK. This might be explained by the training and vigilance of the club’s first aiders observing all matches for concussion. Future studies should try to explain this high rate and subsequently reduce these concussions.

# Abstract: O08: The development of the MaRooN Health Passport: a novel medical platform to identify lifestyle-related alerts in the staff and student population of Stellenbosch University

**Published:** 2019-01-01

**Authors:** S Crumpton, P Viviers, Lara Grobler, W Derman

**Affiliations:** Institute of Sport and Exercise Medicine, Stellenbosch University, Cape Town, South Africa; Campus Health Service, Stellenbosch University, South Africa; Institute of Sport and Exercise Medicine, Stellenbosch University, South Africa; Campus Health Service, Stellenbosch University, South Africa; Institute of Sport and Exercise Medicine, Stellenbosch University, South Africa; Institute of Sport and Exercise Medicine, Stellenbosch University, Cape Town, South Africa; IOC Research Center, South Africa

## Purpose

Chronic non-communicable disease constitutes a rapidly escalating health burden in the South African population, thus there is a growing need to identify risk profiles in the young population to be able to modify these profiles with appropriate interventions. To determine the feasibility of implementation and ascertain risk profiles in young students, the MaRooN (Maties Risk of Non-communicable disease) Health Passport was developed to collect self-reported data.

## Methods

A pilot study was conducted on 600 staff and students from Stellenbosch University to assess the feasibility of the data collection tool, and to determine its ability to identify lifestyle-related risk behaviours and medical conditions. The MaRooN Health passport survey was created on an electronic platform, RedCapR and comprises three sections: (1) demographics, (2) lifestyle behavioural factors, and (3) medical history. A selection of previously validated questionnaires was included in this survey. The link to the survey was distributed to the pilot sample via email for completion.

## Results

381 of 600 participants (64%) accessed the survey, with 234 completing the full survey (61.4% completion rate). 60% of participants were female, and 40% male. The average age was 28 years old (± 10 years; minimum age 18, maximum age 60). 64.2% of participants were students, with 79.7% being undergraduate and 20.3% postgraduate students. The staff participants (33.8%) were classified as academic staff (8.1%) or professional support staff (91.9%). Both lifestyle-related risk behaviours and medical conditions or risks were identified in 54.6% of participants. 15% of participants had only lifestyle-related risk behaviours, whereas 1.3% had only medical conditions or risks. There were no risks identified in 29.1% of the participants. Reasons for non-completion of the survey were identified and the tool amended accordingly. Important data were determined regarding incentivising participants.

## Conclusion

The results of this study suggest that the MaRooN Health Passport is able to effectively identify risk profiles in staff and students. This pilot study has also allowed for effective alteration of the survey and to understand factors relating to survey completion.

# Abstract: O09: Incidence and etiology of volleyball injuries in Ethiopia: a search for amhara regional state clubs and young players

**Published:** 2019-01-01

**Authors:** ET Desalegn, H J van Heerden

**Affiliations:** 1 University of Gondar, College of Natural and Computational Sciences, Department of Sport Science, Gondar, Ethiopia; 2 University of KwaZulu-Natal, School of Health Sciences, Department of Biokinetics, Exercise and Leisure Science, Academic Leader: Research, South Africa, Durban

## Introduction

In recent years in Ethiopia, there is an increment of the number of participants in volleyball sport under clubs, projects and recreational programs. However, athletes are highly vulnerable for injuries because of a lack of protective equipment, suitable playing areas, medical facilities, efficient injury surveillance systems and skilled manpower (coaches).The aim of this study was to examine the incidence and mechanisms of acute and overuse volleyball injuries in Ethiopia and to describe factors associated with injuries.

## Methods

The injury incidence rate, severity, diagnosis and anatomical location of the injuries that occurred during practice and competition in a full season of 40 weeks were recorded prospectively on a weekly basis during the period 2015/16. A total of 323 volleyball players (168 female and 155 male) from Amhara Regional State Clubs and youth teams were participated in the study. All players responded to a questionnaire on demographic variables (only at baseline), sports participation, use of preventive measures, and previous injuries and conducted baseline functional tests (balance, power and flexibility). Volleyball exposure during training and matches was recorded for each individual player by the coach on a weekly exposure form. In the case of an injury triage nurses interviewed the injured player and recorded it on an injury registration form on a weekly basis.

## Results

In the current study, the overall mean age of the players was 15.97 ± 2.94 years for men and 15.86 ± 2.33 years for women. In terms of team type, the age of youth players was 15.42 ± 1.92 years for females and 14.85 ± 1.05 years for males, and club players was 18.54 ± 2.81 years for females and 21.54 ± 2.98 years for male players. During the period of the study (10 months) the total sample (n=323) reported 188 injuries. It is notable that, injury rate in relation to age, the 323 players were divided into three age-related groups. Of the 131 youth and club injured players, 118 (62.77%) players aged 14–16 years, 65 (34.57%) players aged 17–25 and 5 (2.66%) players aged more than 25. The injury rate in the age range of 14–16 players was statistically different in comparison to the injury rate to the other age groups (χ^2^=5.89, p≤0.05). Both youth and club players, reported 188 injuries during a total exposure time 50 379 player-hours, comprising 43 566 hours of training (7 715 hours for club and 35 851 hours for youth players) and 6 813 hours of match play (1 004.5 hours for club and 5 808.5 hours for youth players). The overall injury incidence was 3.73 injuries/1000 player-hours, with 4.31 injuries/1000 player-hours for males and 3.26 injuries/1000 player-hours for females. Regarding team type, 7.1 injuries/1000 player-hours was for club players and 3.02 injuries/1000 player-hours for youths. During match play the injury incidence was 27.59 per 1000 player-hours and 4.31 per 1000 player-hours throughout training periods. The rate of injury in game sessions was statistically different in comparison to training injury rate (χ^2^=16.1,p≤0.05). The result also revealed the rate of injury in game session was significantly different in club and youth players (χ^2^=9.14,p≤0.005) but there were no differences in relation to gender in both game and training sessions. During the season 167 (88.83%) were acute injuries and 21(11.17%) overuse syndromes. According to physician diagnosis, the most common injury was the fingers (55 cases, 29.25%), followed by ankle (35 cases, 18.61%), knee (33 cases, 17.55%). Significant difference was obtained between the anatomical parts (p≤0.05) and gender (p≤0.005). The severity of injuries was shown in terms of absence from competition or training after the injury. The rate of mild injuries was 53.19 % (100 cases), that of moderate injuries was 36.70 % (69 cases), and that of major injuries was 10.11 % (19 cases) (significant difference between them, χ^2^=35.18, p≤0.05). Statistical differences between severities of injury in relation to gender was observed (female χ^2^=18.6,p≤0.05 and male χ^2^=17.19,p≤0.05). Regarding the factors of injury, 57.45% (108 cases) was due to poor technique, 14.89% (28 cases) because of incorrect sprawls, 7.98% (15 cases) caused by stepping on others’ feet and 8.51% (16 cases) as a result of fatigue and significal difference was obtained between the factors of injuries (p≤0.05) but not gender (p>0.05).

## Conclusions

This is the first large scale study of its kind in Ethiopia and has established the epidemiology of injury related to volleyball. Overall, the findings of this study revealed that the frequency of injury was high (58.2%) in the 2015/16 season of volleyball played. In this study male players were more injured than females 51.6% (97/188) and 48.4 % (91/188) respectively. But significant difference was not found between gender (p>0.05). Interms of team type significantly youth players 126 (67.02%) were more injured than elites 62 (32.98%) (p≤ 0.05). Injuries in both groups gender and team type were much more likely to occur in games than in practice. But, significance difference was found only between club and youth players (p=0.05). Finger (29.25%), ankle (18.62%) and knee (17.55%) were the most frequent injured anatomical parts and significant difference was found between the overall anatomical sites (p≤0.05). Sprain was clearly the most common injury in volleyball which accounted nearly about one third 31.38% (59/188) of all injuries with an incidence of 1.17 per 1000 player-hours followed by dislocation and laceration 21.27% (40/188) with an incidence of 0.79 per 1000 player-hours each and statistically significant between the type of injuries diagnosed (p≤0.05) but no significant difference between male and female players (p>0.05). In this prospective study, 10.64% (20 cases) left/right front and 11.7% (22 cases) back line club players and 19.68% (37 cases) back court and 17.55% (33 cases) center young players were vulnerable to injuries due to poor technique 57.45% (108 cases), incorrect sprawl 14.89% (28 cases), fatigue 8.5% (16 cases) and steps on other’s feet 7.98% (15 cases) during the execution of blocking, setting a ball for spikers and receiving first ball. Statistically, significant difference was not obtained between players court position (p>0.05). In relation to degree of severity, more than half 53.19 % (100 cases) injuries were mild severe, 36.7% (69 cases) moderate and 10.1% (19 cases) were major severe. In this regard significance difference was found (p≤0.05).

## Recommendations

In order to prevent players from musculoskeletal injuries, specific warming up, strength, proprioceptive, plyometric, balance and flexibility exercises for the muscles and joints of the upper and lower extremities of players should be incorporated in the training sessions. In addition, players should use preventive equipment during training and match periods. Moreover, continuous injury surveillance and intervention is needed.

# Abstract: O10: Physical activity practice pattern among undergraduate students in the Faculty of Health Sciences

**Published:** 2019-01-01

**Authors:** Adiele Dube, Morgan P. Gundani

**Affiliations:** 1 Department of Health Education, Southern Africa Nazarene University, eSwatini; 2 Department Sports Science & Coaching, National University of Science & Technology, Zimbabwe

## Introduction

Globally, research has revealed serious risks associated with physical inactivity can which include cause deaths worldwide; coronary heart diseases, diabetes, and colon or breast cancer. This study sought to compare the pattern of physical activity practice, benefits and perceived barriers of physical activity among first year and final year undergraduate students.

## Methods

A cross-sectional study was conducted from the Southern Africa Nazarene University’s Faculty of Health Sciences. A total of 480 students were drawn from the 4 departments; Nursing and Midwifery, Medical laboratory sciences and Pharmacy. Physical activity was measured using the International Physical Activity Questionnaire (IPAQ). Descriptive statistics and bivariate analysis; Pearson chi-square test, Fisher exact test were employed for data analysis using SPSS v23.0.

## Results

Sedentarism were predominantly affecting females in the 4^th^ years compared to their 1^st^ year counterparts (p= 0.001). More than 15% of the male students from 1^st^ and 4^th^ years were physically inactive. Time limitation and lack of accessible and suitable sporting facilities were identified by both classes as the most barriers to physical activity (p= 0.001). 83% of the participants stated that physical activity promotes and maintains health. The results showed the correlation between physical activity and sitting time (p = 0.001).

## Conclusion

The main risk groups for physical inactivity were female students. The University should incorporate active physical health lifestyle in the curriculum in order to increase levels of physical activity during medical school training.

# Abstract: O11: Does maturation phase affect physical performance and anthropometry in girls?

**Published:** 2019-01-01

**Authors:** Kirsty Elliott, James Clark, Charne Scott, Cassidy Grobler, Mariska Fisher, Charl Janse van Rensburg

**Affiliations:** 1 Sport, Exercise Medicine and Lifestyle Institute (SEMLI), Faculty of Health Sciences, University of Pretoria; 2 Division of Biokinetics and Sports Science, Department of Physiology, University of Pretoria; 3 Department of Sport and Leisure Studies, University of Pretoria; 4 Biostatistics Unit, South African Medical Research Council

## Introduction

There is limited research on how maturation during stages of puberty affects anthropometry and physical performance in active females. Therefore, the aim of the study is to compare anthropometric measures and physical performance in active females at different maturation stages during puberty.

## Methods

118, physically active females (age: 11.9 ± 0.8 yrs.), were classified into 3 groups according to maturity status (Mirwald equation): Pre-peak height velocity (pre-PHV; n=37), Peak height velocity (PHV; n=52) and Post-peak height velocity (post-PHV; n=29). Chronological age, anthropometry (height, weight, body fat %, BMI (body mass index), arm span, seated height) and performance tests (grip strength, vertical jump, prone bridge, modified sit and reach) were compared between the groups, using a one-way ANOVA.

## Results

Significant (p=0.05) between-group differences were found for chronological age (pre-PHV 11.05 ± 0.52; PHV 11.90 ± 0.50; post-PHV 12.79 ± 0.41 yrs.) and all anthropometric measures (height, weight, body fat %, arm span and seated height) except for BMI (pre-PHV=17.6 ± 2.7; PHV group=19.0 ± 2.4; PHV=19.02 ± 2.4; post-PHV=21.0 ± 2.8; p=0.0001) For performance tests: absolute isometric handgrip strength was significant between maturation groups, (pre-PHV: 18.20 ± 3.96 Right, 17.00 ± 3.80 Left; post-PHV: 28.05 ± 5.79 Right, 25.76 ± 5.32 Left (kg)(p=0.0001)), but strength relative to body weight was not significant (R: p=0.2933 and L: p=0.4611)

## Conclusions

The main finding is that, as females mature during the stages of puberty, there are anthropometric changes (larger body size), but not concomitant changes in physical performance. Therefore, practitioners could group pubertal girls of similar body size together to compete and train with one another in activities that don’t require absolute strength.

# Abstract: O12: Improvements in cardiovascular efficiency over 24-weeks of rehabilitation using robotic locomotor training in persons with spinal cord injury (SCI)

**Published:** 2019-01-01

**Authors:** Robert Evans, Claire Shackleton, Sacha West, Laurie Rauch, Wayne Derman, Yumna Albertus

**Affiliations:** 1 Division of Exercise Science and Sports Medicine, Department of Human Biology, Faculty of Health Science, University of Cape Town, Cape Town, Western Cape, South Africa; 2 Department of Sports Management, Cape Peninsula University of Technology, Cape Town, Western Cape, South Africa; 3 Institute of Sport and Exercise Medicine, Division of Orthopaedic Surgery, Faculty of Medicine and Health Sciences, University of Stellenbosch, Cape Town, South Africa; 4 IOC Research Centre, South Africa

## Research Objective

Evaluate changes in cardiovascular efficiency and walking economy during robotic locomotor training over a 24-week intervention in persons with SCI.

## Design

Single group, repeated measures design.

## Setting

Therapy & Beyond Rehabilitation Centre - Walking with Brandon Foundation

## Participants

Volunteer sample of 8 participants, aged 19 – 60 years, with chronic (>1 year) traumatic motor incomplete SCI (C1–C8; ASIA C-D).

## Interventions

The RLT intervention involved 60-minute sessions, three times per week, over 24-weeks. RLT exclusively used the Ekso GT exoskeleton.

## Main Outcome Measures

Heart rate - HR (beats/min), Six-minute walk test – 6MWT (m) and Total heart beat index – THBI (beats/m).

## Results

Mean HR whilst performing the 6MWT did not vary significantly over the course of the intervention. The distance walked during the 6MWT increased significantly from baseline (68.3 ± 11.3m) to 6-weeks (83.5 ± 8.1m), 12-weeks (98.3 ± 7.5m) and 24-weeks (109.9 ± 19.7m) (p < 0.05). The increased distance walked at 6-weeks with a HR comparable to baseline values, resulted in an improvement in the THBI from 11.1 ± 2.6 at baseline to 7.5 ± 2.8 beats/m walked at 6-weeks (p < 0.05). This improved efficiency of walking stabilised and was maintained from 6 to 24-weeks.

## Conclusions

After 6-weeks of RLT, participants were able to walk greater distances during a 6MWT without increasing their heart rates from baseline values, resulting in a lower THBI. Improved walking efficiency can be attributed to two factors, firstly an improved economy of walking via a learning effect with the exoskeleton and secondly improved cardiovascular fitness due to the aerobic training involved.

# Abstract: O13: Weekly and seasonal patterns of daily wellness monitoring of the 2019 Varsity Cup winners

**Published:** 2019-01-01

**Authors:** Lindsay Starling, Esme Jordaan, Tanya Green, Pierre Viviers, Wayne Derman, James C Brown

**Affiliations:** 1 Institute of Sport and Exercise Medicine, Division of Orthopaedics, Department of Surgical Sciences, Stellenbosch University, Cape Town, South Africa; 2 IOC Research Centre, South Africa; 3 Biostatistics Unit, Medical Research Council, Cape Town, South Africa; 4 Statistics and Population Studies Department, University of the Western Cape, Cape Town, South Africa; 5 Campus Health Service, Stellenbosch University, South Africa

## Introduction

Athlete monitoring is particularly important in university student-athletes who have academic stressors additional to their training. The Stellenbosch Rugby Football Club (Maties) team take part in a prestigious university competition, the Varsity Cup, between the start of February and mid-April of each year. The Varsity Cup competition consists of matches every Monday night and thus teams have fairly consistent weekly training and travel plans. Thus, the aim of this study was to assess if the team displayed similar weekly patterns in their wellness measurements over the period of the competition.

## Methods

During the 71 days of Varsity Cup, the 43 squad members to respond daily on a 5-point Likert Scale for sleep quality, overall stress levels, recovery, body soreness, readiness to train. The patterns in the individual responses for the stressors versus time were inspected, smoothing the data using nonparametric penalized B-splines. Subsequently, the data was grouped into days of the week and generalized linear modelling compared the daily averages for each stressor, incorporating a correlation for the players. Overall and pairwise test results were provided.

## Results and conclusion

Overall, 74% of all possible daily responses were completed by the squad. For all stressors (sleep quality, recovery, body soreness, readiness to train) except overall stress levels, there was an overall difference in mean scores for the weekdays (p<0.001). Specifically, most of the average scores for Monday and Sundays were significantly higher compared to the other days of the week (p<0.05), indicating a clear weekly pattern. There was no weekly pattern for overall stress, but the fitted spline indicated a pattern over the season. In conclusion, these descriptive analyses indicate that monitoring of sleep quality, recovery, body soreness, readiness to train might be sensitive to within-week changes, but that overall stress might be more useful indication of response over the entire competition period.

# Abstract: O14: Health Perception of students from the MaRooN Health Passport at Stellenbosch University: Results from the SF36 questionnaire

**Published:** 2019-01-01

**Authors:** Lara Grobler, Pierre L. Viviers, Wayne E Derman

**Affiliations:** 1 Institute of Sport and Exercise Medicine, Stellenbosch University, South Africa, IOC Research Center, South Africa; 2 Campus Health Service, Stellenbosch University, South Africa

## Purpose

It is prudent that the NCDs and lifestyle risks in the South African student population be described, in order for targeted intervention to be timeously implemented. The aim of the current study was to investigate the perceived wellbeing of a sample of the Stellenbosch student population by means of the SF36 short questionnaire.

## Methods

This study forms part of the MaRooN Health passport, which constitutes a larger and more complete survey covering demographics, lifestyle and behavioural factors and medical history. The survey was distributed to students on Stellenbosch University campus between October 2018 and July 2019. Students completed the survey on an electronic platform, RedCap. Scores range between 0 and 100 with higher scores indicating better perception of health better scores. Sub-categories physical functioning, social functioning, role limitations (physical problems), role limitations (emotional problems), mental health, energy/vitality, pain and general health perception was calculated from the results.

## Results

The survey was completed by 174 students (77 men and 97 women) with a mean age of 21 years (range: 18 years to 29 years). Overall the men and women scored similarly in all sections of the SF36. The lowest average score was found for energy/fatigue levels (women = 50±17, men = 59 ± 14), whereas the highest score was found for physical function (women = 95±14, men = 94 ± 19).

## Discussion

The findings of this study suggest i) the general well-being of the student population is described as “good” and is similar to that described for an age matched population previously described. and ii) low scores for sections relating to energy/fatigue, role limitations due to emotional health, and mental health is of concern and should be addressed in future interventions.

# Abstract: O15: Incidence of Exercise Associated Muscle Cramping (EAMC) in “Comrades” runners - a SAFER study in 84 117 race starters

**Published:** 2019-01-01

**Authors:** Handre Hay, Nicola Sewry, Carel Viljoen, Jeremy Boulter, Martin Schwellnus

**Affiliations:** 1 Sport, Exercise Medicine and Lifestyle Institute (SEMLI), Faculty of Health Sciences, University of Pretoria, South Africa; 2 International Olympic Committee (IOC) Research Centre, South Africa; 3 Sport, Exercise Medicine and Lifestyle Institute (SEMLI) and Department of Physiotherapy, Faculty of Health Sciences, University of Pretoria, South Africa; 4 Medical director, Comrades Marathon

## Introduction

Exercise associated muscle cramping (EAMC) is a common clinical syndrome that requires medical attention during and after endurance running events. The incidence of EAMC during the 90km “Comrades” marathon has not been reported. The aim of this study was to document the incidence of EAMC in runners participating in the “Comrades” ultramarathon race (2014–2018).

## Methods

A retrospective clinical audit, involving 84 117 race starters, was conducted from data collected during the 90km “Comrades” ultramarathon race in South Africa. Medical encounters (MEs) (moderate and serious life-threatening), were collected by medical doctors during and immediately after the event. Data were retrospectively coded using the 2019 consensus statement definitions. The main outcome variables were: overall crude incidence rate (IR per 1 000 starters; 95% CIs) for EAMC, and prevalence (as a % runners with EAMC) of symptoms of more severe EAMC.

## Results

The overall IR of EAMC over the five year period was 3.1 (95% CI: 2.7 – 3.5) (n=259) (1 in 322 race starters). The crude IR varied by year, with the lowest IR reported in 2015 (IR=1.7; 95% CI: 1.1 – 2.3) and the highest reported in 2017 (IR=4.7; 95% CI: 3.7 - 5.7). The incidence of more severe EAMC over the five years was 1.7 (95%CI: 1.4 – 2.0). The three most common presenting symptoms of more severe EAMC (n=144) were associated nausea/vomiting (66%), fatigue/exhaustion (36%) and dizziness (13%).

## Conclusions

The incidence of more severe EAMC in runners participating in the 90km “Comrades” ultramarathon (90km, IR=1.7), is double that compared to data from the 21.1km and 56km Two Oceans races (IR=0.91). Factors associated with this high incidence require investigation, so that prevention strategies can be designed and implemented to manage the risk of EAMC in “Comrades” runners.

# Abstract: O16: Cardiac rehabilitation delivery in Africa

**Published:** 2019-01-01

**Authors:** Martin Heine, Karam Turk-Adawi, Marta Supervia, Wayne Derman, Pamela Naidoo, Sherry Grace

**Affiliations:** 1 Institute of Sport and Exercise Medicine, Faculty of Medicine and Health Sciences, Stellenbosch University; 2 Division of Physiotherapy, Faculty of Medicine and Health Sciences, Stellenbosch University; 3 IOC Research Centre, Cape Town, South Africa; 4 Qatar University, Doha, Qatar; 5 Gregorio Maranon Health Research Institute, Gregorio Maranon General University Hospital, Madrid, Spain; 6 Division of Preventive Cardiology, Department of Cardiovascular Medicine, Mayo Clinic, Rochester, USA; 7 Heart and Stroke Foundation, Cape Town, South Africa; 8 African Heart Network, Cape Town, South Africa; 9 Department of Psychiatry, Faculty of Health and Medicine, Stellenbosch University, Cape Town, South Africa; 10 York University, University Health Network, Toronto, Ontario, Canada

## Introduction

The burden of non-communicable diseases (NCDs), and cardiovascular disease (CVD) specifically, is growing exponentially in Africa, at a rate exceeding that globally. Cardiac rehabilitation (CR) is an established model of care designed to mitigate the burden of NCDs, and CVD specifically. Robust evidence from predominantly high-income countries demonstrates that CR is associated with a reduction in rates of cardiovascular mortality (by 26%) and rehospitalisation (by 18%), as well as increases in quality of life, while being cost effective.

## Methods

Availability, capacity and characteristics of CR services around the globe were evaluated through a first-ever international survey. Where available, champions from each country with CR were enlisted to identify the number of programmes, and also to administer an online survey of these programmes, which assessed both patient capacity and programme characteristics. CR density was computed using Global Burden of Disease study estimates for annual Ischemic Heart Disease (IHD) prevalence, to characterise the number of CR spots available per patient in need per year.

## Results

A total of 32 CR programmes were identified across 8/47 (17%) African countries (the lowest proportion of all WHO regions). Raw unmet need IHD alone (considering zero availability in countries without CR) for Africa was 1 383 858 spots. Eighty percent of African programmes fulfilled the 20 structure and process indicators (e.g. assessment of risk factors) which were assessed in the survey.

## Conclusions

These findings, particularly in the context of the projected accelerated increase in incidence of NCDs in Africa demonstrate CR capacity must be augmented massively. Given the realities of the African context (e.g. lack of trained healthcare professionals, limited resources, geographical challenges, multi-faceted and complex burden of disease), how to do this feasibly remains to be determined.

# Abstract: O17: More than 70% of employees in a South African financial institution have modifiable risk factors for non-communicable disease (NCD) – Project SWAY

**Published:** 2019-01-01

**Authors:** Nceba Hene, Martin Schwellnus, Paola Wood

**Affiliations:** 1 Sport, Exercise Medicine and Lifestyle Institute (SEMLI) and Division of Biokinetics and Sports Science, Department of Physiology, Faculty of Health Sciences, University of Pretoria, South Africa; 2 Sport, Exercise Medicine and Lifestyle Institute (SEMLI) and Section Sports Medicine, Faculty of Health Sciences, University of Pretoria, South Africa; 3 International Olympic Committee (IOC) Research Centre, South Africa

**Keywords:** Non-communicable diseases, modifiable risk factors, BMI, physical activity, chronic disease, modifiable risk factors

## Purpose

Non-communicable diseases (NCDs) currently accounts for most deaths in South Africa (55% of all deaths). Understanding the prevalence of modifiable risk factors for NCDs in a sub-population is vital to guide the planning of any future intervention programme to reduce the risk of NCD. The aim of this study was to determine the prevalence of modifiable NCD risk factors in employees of a privately funded health insurer in the financial sector in order to plan and implement a social media based NCD intervention programme (Project SWAY).

## Methods

In this descriptive observational cross-sectional analysis, data from 10655 employees (38.3% male; 61.7% female: mean age ± SD = 48.1 ± 11.5 yrs) were analysed. NCD risk factor data were obtained from health risk assessments that included self-reported questionnaire (demographics, medical history, physical activity and current smoking status) and clinical measures (height, mass, BMI, total cholesterol, random glucose, and resting blood pressure). The main outcome measure is the prevalence of NCD risk factors (% of employees).

## Results

The prevalence of modifiable NCD risk factors were: consumption of <3 fruit/vegetable portions/day (88.7%); inadequate physical activity (75.9%; employees performing <150 minutes of moderate-intensity physical activity/week); BMI >25 (71.4%; overweight =33.8%, obese=36.8%); raised resting systolic (34.3%; >130 mmHg) and diastolic (52.1%; >90mmHg) blood pressure; cigarette smoking (18%) and raised total cholesterol (14%; >5.2 mmol/l). The prevalence of these risk factors was similar for males and females.

## Conclusion

Poor nutritional habits, inadequate physical activity and overweight/obesity are the principal modifiable NCD risk factors (>70% of the studied population). A planned social media based NCD intervention programme (Project SWAY) to reduce lifestyle risk factors in this population should mainly target these three modifiable NCD risk factors.

# Abstract: O18: Unilateral transtibial amputees are most satisfied with a prosthesis that utilises a novel linkage system: a randomised controlled trial

**Published:** 2019-01-01

**Authors:** Nicole Hinze, Elton Wayne Derman, Phoebe Runciman

**Affiliations:** 1 Institute of Sport and Exercise Medicine, Department of Orthopaedics, Stellenbosch University; 2 Institute of Sport and Exercise Medicine, Department of Sport Science, Stellenbosch University

## Objectives

This study describes the subjective ratings of prosthesis satisfaction and daily fatigue in a group of South African unilateral transtibial amputees (UTTAs) using a conventional solid-ankle cushioned-heel (SACH) foot, a Vari-Flex energy storage and return (ESAR) foot and a novel linkage system Pro-Flex ESAR foot.

## Methods

Twenty UTTAs performed a standardized protocol using a different foot prosthesis every two weeks, in a double-blind, randomised cross-over order. After two weeks of acclimatisation, the participants completed the Trinity Amputation and Prosthesis Experience Scales Revised (TAPES-R) and Patient-Reported Outcome Measurement Information System (PROMIS) questionnaires, which are validated, and use a seven-day recall period. The TAPES-R questionnaire measures the subjective experience of wearing a particular prosthesis. The multiitem PROMIS questionnaire measures the participants’ fatigue and subsequent effects on everyday living.

## Results

The UTTAs in this study wore each prosthesis for 13 ± 3 hours per day. The UTTAs rated the novel Pro-Flex as significantly more comfortable (p = 0.031), useful (p = 0.00027) reliable (p = 0.0096) and satisfactory (p = 0.00013) compared to the SACH, with also a higher rating in comfort (p = 0.55), usefulness (p = 0.51) and reliability (p = 0.68) than the Vari-Flex Foot. UTTAs reported experiencing more fatigue which interferes with social activities (p = 0.034), task completion (p = 0.03), task initiation (p = 0.041) and self-efficacy (p = 0.029) whilst using the SACH, compared to the Vari-Flex. However, UTTAs wearing the Pro-Flex reported an overall higher fatigue (p = 0.89) than when wearing the Vari-Flex.

## Conclusion

The current study shows that UTTAs prefer the novel Pro-Flex foot over the Vari-Flex and SACH (p < 0.05) foot. However, it is interesting to note that the Pro-Flex caused more fatigue in daily life than the Vari-Flex, which may be as a result of the increased energy required to control the more deformable Pro-Flex foot, due to the novel foot/ankle linkage system. More research is required to investigate this relationship.

# Abstract: O19: Is the risk of injury in a Super Rugby season increased by injury in the preceding season?

**Published:** 2019-01-01

**Authors:** Charl Janse Van Rensburg, Nicola Sewry, Martin P. Schwellnus, Esme Jordaan

**Affiliations:** 1 Sport, Exercise Medicine and Lifestyle Institute (SEMLI), Faculty of Health Sciences, University of Pretoria, Pretoria, South Africa; 2 International Olympic Committee (IOC) Research Centre, South Africa; 3 Emeritus Professor of Sport and Exercise Medicine, Faculty of Health Sciences, University of Cape Town, South Africa; 4 Biostatistics Unit, Medical Research Council, Parow, South Africa; 5 Statistics and Population Studies Department, University of the Western Cape

## Introduction

Rugby union is a contact sport resulting in injuries, which may increase the risk of future injury. This study aimed to investigate whether a player who sustained an injury in a previous season, had a higher risk of sustaining an injury in the following season.

## Methods

A prospective observational study in 686 elite South African rugby player seasons in the Super Rugby Tournament (2014 – 2016). Team physicians daily reported injuries sustained during match and training throughout the tournament (58 789 player-hours). To indicate the playing characteristics in the previous season 2 variables were coded to indicate if the player (1) played (PPS), (2) was injured (IPS) in the previous season. The model included season, PPS, IPS, season*IPS, match and training hours for both previous and current season, and lastly position played in current season (forward/back). The incidence rates (IR; 95%CI) are reported for the IPS groups (IPS positive and IPS negative).

## Results

Out of the 102 (2014), 109 (2015) and 109 (2016) with injuries in the current season, 40 (39%), 46 (42%) and 41 (38%) also sustained injuries in the previous season. The IR for the IPS positive and negative groups were 10.7 (8.6–13.2) and 9.4 (8.2–10.8), which did not differ significantly. There was not a significant difference between the IRs for forwards and backs, however the IR increased significantly for an increase in total match hours in current season, and decreased significantly for an increase in total training hours in current season (p<0.0001). Player hours in previous season is marginally significant (p=0.041). The IPS*season interaction was significant (p=0.021), with a significant difference only between 2014 and 2016 (p=0.006).

## Conclusion

After adjusting for various factors, whether a player had previous injury did not increase their injury incidence rate for the current season. However, player load both in current and previous season may be important predictors. Further investigation is required.

# Abstract: O20: Measures to reduce illness burden during the Super Rugby Tournament should focus on preventing infective respiratory tract illness – A prospective study involving 103979 player days

**Published:** 2019-01-01

**Authors:** Audrey Jansen van Rensburg, Martin P Schwellnus, Dina C (Christa) Janse van Rensburg, Esme Jordaan, Charl Janse van Rensburg

**Affiliations:** 1 Sport, Exercise Medicine and Lifestyle Institute (SEMLI) and Section Sports Medicine, Faculty of Health Sciences, University of Pretoria, Pretoria, South Africa; 2 International Olympic Committee (IOC) Research Centre, South Africa; 3 Emeritus Professor of Sport and Exercise Medicine, Faculty of Health Sciences, University of Cape Town, South Africa; 4 Biostatistics Unit, Medical Research Council, Parow, South Africa; 5 Statistics and Population Studies Department, University of the Western Cape

## Introduction

Respiratory tract illness (RTI) accounts for most medical consultations in elite athletes, but details of the type, clinical diagnoses and severity are lacking. This study aims to describe the incidence, diagnoses (clinical, specific) and illness burden of RTI in rugby players during the annual Super Rugby tournament.

## Methods

Elite rugby players (n=1141) from 5 South African teams were followed over the 16-week competition period for each year 2013–2017 (103979 player days). Team physicians daily completed an illness log, with squad size and RTI details: clinical diagnoses (infective/non-infective), specific diagnoses, and training/match days lost to RTI. Incidence rates (IR) (per 1000 player days; 95%CI) of RTI (all, infective, non-infective) and specific diagnosis, illness burden (IB) (days lost/1000 player days) and days lost per single illness (days; 95%CI) were reported.

## Results

RTI accounted for 67.7% of all illness. The IR of infective RTI (2.6; 2.3–3.0), was higher than non-infective RTI (0.4; 0.3–0.6) (p<0.001). Specific diagnoses was: 1) infective RTI - acute upper RTI (2.0; 1.8–2.3; 45.4%), influenza (0.2; 0.1–0.3; 4.5%), acute lower RTI (0.1; 0.1–0.2; 2.6%); 2) non-infective RTI - allergic rhinitis (0.2; 0.1–0.3; 3.6%), allergic sinusitis (0.2; 0.1–0.2; 3.4%). The IB of infective RTI (3.3; 2.9–3.6), was significantly higher than non-infective RTI (0.9; 0.7–1.1) (p<0.05). The IB for specific infective RTI was highest for acute infective upper RTI (2.0; 1.8–2.3), followed by influenza (0.4; 0.3–0.6), and acute infective lower RTI (0.4; 0.3–0.5). Days lost per single illness was highest for pneumonia (15.0; 8.4–24.7), acute infective lower RTI (3.2; 2.2–4.4) and influenza (2.1; 1.5–2.8).

## Conclusion

Infective RTI accounted for >58% of all illness, mostly presenting as acute infective upper RTI (45.4%) resulting in the highest IB. However, days lost per single illness was highest for pneumonia and acute infective lower RTI. Measures to reduce the IB of RTI should focus on preventing infective RTI.

# Abstract: O21: There is a non-linear relationship between total annual training distance and overuse injuries in cyclists: A cross-sectional study in 21617 recreational cyclists

**Published:** 2019-01-01

**Authors:** Esme Jordaan, Martin Schwellnus, FC Du Toit, Jannelene Killops

**Affiliations:** 1 Biostatistics Unit, South African Medical Research Council; Statistics and Population Studies, University of the Western Cape; 2 Sport, Exercise Medicine and Lifestyle Institute (SEMLI), Faculty of Health Sciences, University of Pretoria; International Olympic Committee (IOC) Research Centre, South Africa; 3 Sport, Exercise Medicine and Lifestyle Institute (SEMLI) and Division of Biokinetics and Sports Science, Department of Physiology, Faculty of Health Sciences, University of Pretoria, South Africa

## Introduction

Training load is a risk factor associated with overuse injuries, but training load and overuse injuries in cyclists has not been well investigated. The aim of this analysis was to explore the relationship between annual self-reported training distance and overuse injuries in recreational cyclists participating in a mass community-based cycling event.

## Methods

This cross-sectional study included data from the pre-screening medical questionnaire completed by 21 617 consenting race entrants for the 2016 Cape Town Cycle Tour, including demographics (age, sex), training variables the last 12 months (km/week, frequency of training per week, training speed), and overuse injuries in the past 12 months. 615 cyclists reported 644 overuse injuries. Using the training data variables, an estimated total training distance (TTD) in the past 12 months (km) was calculated (reported weekly average distance x 52 weeks). The relationship between TTD (km) and injury rate (IR: % cyclists with overuse injury) was explored using non-parametric penalized B-spline curves and the generalized linear model.

## Results

The overall overuse incidence rate was 2.8 (95%CI: 2.6–3.1). The average TTD was ≈5000km per year. There was a significant non-linear relationship between TTD and overuse IR (p<0.01), with a relatively low IR (<3%) at TTD below 5 200km/year, a steady increase in IR up to a peak IR (4%) at TTD of 14 000km/year, and a decrease in IR at TTD > 16 600km/yr. There is a consistently higher IR for females compared to males over the range of TTD (p=0.012). There is a consistently higher IR for older cyclists (older than 50 years) compared to younger cyclists (50 years and younger) over the range of TTD (p=0.0003).

## Conclusions

These results indicate that an increase in TTD increases the overuse injury rate, but after a point, the rate decreases as TTD increases. Further investigations should be performed to investigate the reason for the eventual decrease in the injury rate.

# Abstract: O22: Exercise and laser therapy for knee osteoarthritis: A Survey to understand the knowledge and attitudes of medical and allied health practitioners

**Published:** 2019-01-01

**Authors:** A Kholvadia, D Constantinou, P J-L Gradidge

**Affiliations:** Centre for Exercise Science and Sports Medicine, Faculty of Health Sciences, University of the Witwatersrand, Johannesburg, South Africa

## Background

There is evidence on the use of modalities such as exercise and Low Level Laser therapy (LLLT) for the management of Knee Osteoarthritis (KOA), and Medical and Allied practitioners play a vital role in prescribing these various modes of management. The purpose of this study was to assess the prescribing tendencies, attitudes and beliefs of South African Medical and Allied practitioners on five modes of modes of KOA management.

## Methods

A convenience sample of 413 Medical and Allied practitioners, including General Practitioners (n=110), Specialist Practitioners (n=117), Allied Practitioners (n=158) and Natural/Homeopathic Practitioners (n=28) participated in this survey method descriptive study. A validated questionnaire was distributed electronically to a data base of practitioners with a 57.12% completion rate after six months of regular contact. Descriptive statistics and frequency tables were calculated. The Pearson’s Chi Square statistic was used for testing the relationship between deemed efficacy and deemed compliance of the pharmaceuticals, surgical interventions, homeopathic intervention, exercise therapy and LLLT. All statistical significance as set at p<0.05.

## Results

The highest percentage of practitioners recommending pharmaceutical or physical exercise therapy. However practitioners viewed the deemed efficacy of pharmacological interventions greater than that of exercise therapy and this trend was followed for deemed compliance of the two modes of therapy. Practitioners (36%) recommended pharmacotherapy with significant efficacy (p = 0.001) and compliance (p = 0.000). However awareness of LLLT as a management tool showed significant efficacy (p = 0.000) and compliance (p = 0.000) despite being recommended by <2% of respondents as a management tool.

## Conclusion

Although medical and Allied practitioners were positive towards the use of exercise in the management of KOA, their prescription tendencies did not always align with the deemed efficacy due to the effect of deemed compliance. Therefore the dissemination of results warrants the gap between educating practitioners on various modes in the management of KOA that addresses the structural, functional aspects of the disorder together with improved efficacy and compliance

# Abstract: O23: A history of multiple chronic diseases is associated with Muscle strain injuries in runners: A cross-sectional SAFER study in 76654 runners

**Published:** 2019-01-01

**Authors:** Lize Kroon, Martin Schwellnus, Paola Wood, Sonja Swanevelder, Esme Jordaan, Kim Nolte

**Affiliations:** 1 Sport, Exercise Medicine and Lifestyle Institute (SEMLI) and Division of Biokinetics and Sports Science, Department of Physiology, Faculty of Health Sciences, University of Pretoria, South Africa; 2 Sport, Exercise Medicine and Lifestyle Institute (SEMLI), Faculty of Health Sciences, University of Pretoria, South Africa; 3 International Olympic Committee (IOC) Research Centre, South Africa; 4 Emeritus Professor, Faculty of Health Sciences, University of Cape Town, South Africa; 5 Biostatistics Unit, South African Medical Research Council; 6 Statistics and Population Studies Department, University of the Western Cape

**Keywords:** Muscle strain injury, recreational running injuries, chronic disease, medication use, intrinsic risk factors

## Purpose

The purpose was to determine risk factors associated with MSI in recreational runners participating in a mass community-based running event 2012–2015 Two Oceans marathon.

## Methods

Online pre-race data from 76654 consenting runners (60.7 % for 21.1 km, 39.4% for 56 km) were analysed, using a cross-sectional observational study design. Risk factors associated with MSI were explored using multi-variate analyses reporting a prevalence ratio (PR) and level of significance (p≤0.05): runner demographics, race distance, training/racing history, medication use, symptoms, history and risk factors of existing chronic disease.

## Results

A total of 2093 (3.34%) entrants reported MSI. When comparing race entrants who reported a MSI to those who did not, older age (>31yrs)(p<0.0001), male sex (PR=1.27; p<0.0001), and longer race distance (56km vs. 21.1km)(PR=2.09; p<0.0001) were associated with a higher prevalence of MSI. Independent risk factors associated with MSI (adjusted PR for race distance, sex and age) were: increased years of recreational running (*p<0.0001*), history of cardiovascular disease (CVD) (PR =1.40; *p=0.0011*), symptoms of CVD (PR =1.49; *p=0.0016*), chronic gastrointestinal tract disease (PR =1.60; *p*<0.0001), respiratory disease (PR =1.48; *p*<0.0001), haemoglobin immune disease (PR=1.50; *p=0.0166*), allergies (PR =1.80; *p<0.0001*), prescribed chronic medication use (PR= 1.14; *p=0.0424*) and history of use of analgesic/anti-inflammatory medication use (PR =4.55; *p<0.0001*).

## Conclusion

Risk factors that should be considered when implementing prevention programs are age, race distance and male sex as well as years participation in endurance running events, medication use and a history and symptoms for chronic disease. Future research must focus on these novel independent risk factors.

# Abstract: O24: Injury surveillance in school rugby union in Ireland

**Published:** 2019-01-01

**Authors:** Therese M. Leahy, Ian C. Kenny, Mark J. Campbell, Giles D. Warrington, Roisin Cahalan, Andrew J. Harrison, Mark Lyons, Liam G. Glynn, Thomas M. Comyns

**Affiliations:** 1 Department of Physical Education and Sport Sciences, University of Limerick, Limerick, Ireland; 2 Health Research Institute, University of Limerick, Limerick, Ireland; 3 Lero, The Irish Software Research Centre, University of Limerick, Limerick, Ireland; 4 School of Allied Health, University of Limerick, Limerick, Ireland; 5 Graduate Entry Medical School, University of Limerick, Limerick, Ireland

## Introduction

Rugby Union has increased in popularity among children and adolescents in recent years. School Rugby in particular often represents a development pathway into elite academy and club Rugby. Rugby is a physically demanding sport and evaluating the injury risk in school players is paramount to player welfare and safety. Injury risk within the school cohort remains unclear which is largely due to wide variations in study methodologies and surveillance practices.

## Methods

The Irish Rugby Injury Surveillance (IRIS) project developed a Rugby specific web-based surveillance system (IRISweb). Injury data were collected from 11 elite senior (Senior Cup) school teams across one season using the IRISweb system. Injury recorders inputted data for 305 male players aged 17–19 years for 95 matches (70mins duration).

## Results

The overall match time-loss incidence rate was 67.8/1,000 player hours. The most common injuries were ankle ligament sprains (17%; 11.4/1,000 player hours), shoulder subluxations/dislocations (11%; 7.2/1,000 player hours) and concussion (10%; 6.6/1,000 player hours). The shoulder was the most commonly injured body location (26%; 17.4/1000 player hours). The majority of injuries occurred during the tackle event (56%), with more injuries resulting from tackling than being tackled. Sprains were the most common injury type. In School Rugby the blindside flanker suffered the most injuries (14%). Most injuries occurred during the third quarter of the match.

## Conclusion

The incidence rates reported here are higher than previously reported (Leahy et al., 2019) for school Rugby and adult amateur Rugby (Yeomans et al., 2018), however, are lower than those reported for the professional game (Williams et al., 2013). Compared to adult amateur rates and locations, schools can seek to protect younger players in target ways. When added to pooled data from future seasons of IRIS injury surveillance, this information aims to enhance the health and welfare of Rugby Union players across the Irish school game and beyond by providing information on injury patterns that can impact on governing policy regarding injury prevention measures.

## References

[b1-2078-516x-31-v31i1a7466] LeahyTM KennyIC CampbellMJ WarringtonGD CahalanR HarrisonAJ LyonsM GlynnLG ComynsTM Injury surveillance in school Rugby: A systematic review of injury epidemiology & surveillance practices Physical Therapy in Sport 2019 38 170 8 3115874110.1016/j.ptsp.2019.05.005

[b2-2078-516x-31-v31i1a7466] WilliamsS TrewarthaG SimonK 2013 A meta-analysis of injuries in senior men's professional rugby union Sports Medicine 43 10 1043e1055 2383977010.1007/s40279-013-0078-1

[b3-2078-516x-31-v31i1a7466] YeomansC Kenny IanC CahalanR WarringtonGD HarrisonAJ HayesK 2018 The incidence of injury in amateur male rugby union: A systematic review and meta-analysis Sports Medicine 48 4 837e848 2929987610.1007/s40279-017-0838-4PMC5856893

# Abstract: O25: Physical activity and risk factors for chronic non-communicable disease in relation to disease severity in patients with Multiple Sclerosis – a cross sectional survey

**Published:** 2019-01-01

**Authors:** Desiree Maartens, Wayne Derman, Susan Hanekom, Martin Heine

**Affiliations:** 1 Institute of Sports and Exercise Medicine, Faculty of Health and Medicine, Stellenbosch University, Cape Town, South Africa; 2 Department of Physiotherapy, Faculty of Health and Medicine, Stellenbosch University, Cape Town, South Africa

## Introduction

Due to the improved medical management of patients with MS (pwMS) there has been an increased interest in secondary prevention of chronic comorbidity. It can be hypothesized that with increasing disease severity, pwMS are less likely to engage in physically activity thereby are predisposed for factors for comorbidity and specifically chronic noncommunicable diseases of lifestyle.

## Methods

The first ever national survey including outcomes on health status, physical activity, and societal participation of patients with MS in South Africa was conducted through the national MS Society. Self-reported risk factors including Body Mass Index (BMI; underweight, normal, overweight, obesity I – III), presence of high blood pressure and high cholesterol, were compared using descriptive statistics across levels of disability (Patient Determined Disease Steps [PDDS]; mild, moderate, severe) and physical activity (International Physical Activity Questionnaire [IPAQ]; Inactive, Minimally-Active, and Health-Enhancing Active).

## Results

122 of 1 000 (12.2%) completed the survey (Age=47.4±9.7yr, Male (%) =14, disease duration=10.7±9.1yr). Patients with moderate MS reported a significant (Chi-Square<.05) higher presence of high blood pressure (37%), and high cholesterol (37%) relative to patients with mild MS (12 and 14%). Independent of disease severity, >68% of pwMS were either overweight or obese. Patients with severe MS reported the highest BMIs (Obese class I 27%, class II 14% and class III 9%). Five percent of pwMS are health-enhancing active, 31% minimally active and 64% inactive. There was a trend (p=.06) towards lower levels of physical activity in patients with moderate MS (Inactive=77%, Minimally-Active=23%).

## Conclusion

Patients with MS are generally inactive independent of disease severity. The presence of self-reported risk factors significantly increased with disease severity, and are indicative of a higher risk for chronic non-communicable disease across the lifespan.

# Abstract: O26: Influence of an exercise program on perceptions and knowledge of modifiable risk factors for non-communicable diseases

**Published:** 2019-01-01

**Authors:** Sweetness J. Makamu-Beteck, Sarah J. Moss, Lainie Cameron, Francois G. Watson

**Affiliations:** 1 Physical Activity, Sport And Recreation, North-West University, Potchefstroom; 2 School Of Health And Wellbeing, University Of Southern Queensland; 3 NUMIQ Focus Area, North-West University, Potchefstroom

## Background

Health behaviours are embedded in knowledge, attitudes, and beliefs about illness and health. Facilitators and barriers need to be understood to promote healthy behaviours. In this study, we explored the influence of an exercise intervention on the perception and knowledge of modifiable risk factors for non-communicable diseases among black women.

## Methods

We used a convergent parallel mixed-method design. Qualitative and quantitative data were collected at baseline (n=95), 12-weeks (n=55), and 24-weeks post-intervention (n=44) from Black African females aged 28 to 78 years. Heart disease and physical activity knowledge surveys were completed at these three time points. Measures of modifiable risk factors for NCDs, namely body fat percentage, Body mass index, blood pressure, peripheral blood glucose, serum cholesterol, and objective physical activity for seven consecutive days, were collected. A subgroup of 25 women participated in focus group discussions exploring their knowledge and perceptions of modifiable risk factors for non-communicable diseases. Participants received a progressive, supervised exercise intervention once per week for 60 minutes during the intervention of 24-weeks.

## Results

The findings indicate that 12-weeks of exercise intervention significantly decrease the systolic blood pressure from 142 to 134 mmHg (p < 0.001); increased physical activity level from 1.4 to 1.6 (p<0.001), physical activity knowledge (8.8 to 9.4; p<0.001) and knowledge of heart disease (11 to 14; p<0.001). BMI decreased significantly from baseline (32 kg/m^2^) to 12-weeks (31 kg/m^2^; p = 0.02) of intervention. Physical activity level, physical activity knowledge, heart disease knowledge, SBP and BMI remained unchanged from 12- to 24-weeks of exercise intervention. An adapted health belief model was used to interpret the qualitative findings. Health exposures and cultural traditions influenced the perceptions of the participants. Increased physical activity levels were driven by motivation from others and experiencing health improvements.

## Conclusions

Supervised exercise has shown to positively influence Black African females’ healthy behaviours through a deeper understanding of cultural perceptions of modifiable risk factors for non-communicable diseases. Structural knowledge of physical activity was not increased by the 24-week supervised exercise, although BP was significantly reduced.

# Abstract: O27: The batting backlift technique in cricket: what is the consensus at all skill levels?

**Published:** 2019-01-01

**Authors:** Habib Noorbhai

**Affiliations:** 1 Department of Sport and Movement Studies, Faculty of Health Sciences, University of Johannesburg, Johannesburg, South Africa

## Introduction

The batting technique in cricket consists of various elements such as the grip, stance, backlift, downswing, impact with the ball and follow through. Whilst there has been an extensive amount of research into these batting elements, there is not an adequate amount of research specifically on the backlift technique. We aimed to investigate and provide a scientific understanding of the batting backlift technique (BBT) in cricket at the various skill levels.

## Aims

We aimed to investigate the BBT of the most successful batsmen (n = 65) in the last 120 years; players in the Indian Premier League (IPL) (n = 30); semi-professional, professional and international cricketers (n = 155) from South Africa and the United Kingdom; and coached and uncoached cricketers (UC) (n = 80).

## Methods

Biomechanical and video analyses were performed on all participant groups. Classifiers were utilised to identify the batting backlift technique type (BBTT) employed by all batsmen. All statistics and wagon wheels (scoring areas of the batsmen on a cricket field) were sourced online. A Pearson’s Chi-squared test, Student T-test and T-test were performed in this study. All analyses were performed using R (R Core Team, 2014) at a significance level of α = 0.05.

## Results

It was found that successful batsmen over the last 120 years did not conform to the current cricket coaching method that advocates a straight batting backlift technique (SBBT). Instead, 77% of successful batsmen and 90% of IPL batsmen employed a lateral batting backlift technique (LBBT) in which they lifted their bats in the direction of second slip or beyond with the bat face towards the off-side. Using this technique, both the toe of the bat and face of the bat points directly towards the off-side (usually between slips and point). The number of players using the LBBT was significantly greater than those using the SBBT (χ2 = 19.2, df = 1, p 0.001). Given these findings, we were curious to determine whether this finding was similar at other levels of cricket. It was found that a LBBT is more prevalent at the highest levels of the professional game and a likely contributor factor for successful batting at the highest level (p0.05). We then proceeded to investigate the BBT amongst the lower levels of cricket (junior and adolescent cricketers). It was found that more than 70% of UC adopted a LBBT, whereas more than 70% of coached cricketers adopted the SBBT.

## Discussion and Conclusions

The LBBT is a likely contributing factor to successful batsmanship at all levels of cricket ability (junior cricketers, adolescent cricketers, semi professional cricketers, professional cricketers, international cricketers and former elite/successful cricketers). Early coaching emphasising the SBBT (one of the basic fundamentals of batting coaching) could be less favourable to the long-term success of young cricketers. Coaching a LBBT to young batsman may be challenging and, therefore, a coaching cricket bat has been developed and has shown to be a promising training aid for coaching the LBBT to young cricketers. The findings and elements represented in this paper are not generalisable to both men and women’s cricket, and further research should be conducted to reach the consensus of the backlift in women’s cricket.

# Abstract: O28: Ligament injuries occur in every match during the Super Rugby Tournament (2013 – 2016)

**Published:** 2019-01-01

**Authors:** Sibusiso Ntuli, A Jansen van Rensburg, Martin P. Schwellnus, Dina C (Christa) Janse Van Rensburg, Charl Janse Van Rensburg, Esme Jordaan

**Affiliations:** 1 Sport, Exercise Medicine and Lifestyle Institute (SEMLI) and Section Sports Medicine, Faculty of Health Sciences, University of Pretoria, Pretoria, South Africa; 2 International Olympic Committee (IOC) Research Centre, South Africa; 3 Emeritus Professor of Sport and Exercise Medicine, Faculty of Health Sciences, University of Cape Town, South Africa; 4 Biostatistics Unit, Medical Research Council, Parow, South Africa; 5 Statistics and Population Studies Department, University of the Western Cape

## Introduction

Rugby union is a contact sport resulting in injuries, mostly to the soft tissues (muscle-tendon and ligaments). There are limited data on the incidence, specific anatomical area affected, severity and mechanisms of ligament injuries in rugby union. This study aimed to determine the incidence rate (all, match and training; specific anatomical area), overall severity and general mechanism of ligament injuries amongst elite rugby players.

## Methods

A prospective observational study in 866 elite South African rugby players participating in the Super Rugby Tournament (2013–2016). Team physicians daily reported injuries sustained during match and training throughout the tournament (76301 player-hours, 6520 match player-hours, 69781 training player-hours). Overall injury rates (IR per 1000 player-hours: 95%CI), IR by anatomical area, severity grade (% moderate/severe: >8 days lost), and mechanism (% contact vs. non-contact) of ligament injuries are reported.

## Results

257 ligament time-loss injuries were recorded (84.1% during matches, 15.9% during training) with an overall IR of 3.4 (3.0–3.8). Ligament IR was significantly higher during matches (33.1; 28.9–37.9) vs. training (0.6; 0.4–0.8) (p<0.001). The IR of match ligament injuries was significantly higher in the lower limb (18.7; 15.5–22.3) vs. the upper limb (9.8; 7.6–12.5) (p<0.05). Specific ligament IR, within main anatomical areas, was highest in the ankle (0.88; 0.68–1.12) and knee (0.86; 0.67–1.10), followed by the shoulder/clavicle (0.59; 0.43–0.79). A total of 44.8% ligament injuries were classified as moderate/severe (>8 days lost), and contact (80.8%) rather than non-contact (19.2%) (p<0.001) was the general mechanism of ligament injuries.

## Conclusion

During the Super Rugby Tournament, >1 ligament injury will occur in every match played by two teams. The most common ligament injuries occur in the lower limb (ankle and knee region) and shoulder/clavicle. In 45% of cases, the player will not be available for training/match for at least 8 days. This information can positively influence prevention programs.

# Abstract: O29: Performance and anthropometrical differences between sexes at the onset of puberty

**Published:** 2019-01-01

**Authors:** Charne Scott, Kirsty Elliott, James Clark, Cassidy Grobler, Mariska Fisher, Charl Janse van Rensburg

**Affiliations:** 1 Sport, Exercise Medicine and Lifestyle Institute (SEMLI) and Division of Biokinetics and Sports; 2 Science, Department of Physiology, Faculty of Health Sciences, University of Pretoria; 3 Department of Sport and Leisure Studies, University of Pretoria; 4 Biostatistics Unit, South African Medical Research Council

## Introduction

Understanding anthropometric and physical performance capability between youths of similar maturational status (pre-pubertal) but in different sexes, is needed to guide professionals in the prescription of exercise and sport. The aim of this study was to compare anthropometric and physical performance parameters in pre-pubertal (pre-peak height velocity; pre-PHV) girls and boys.

## Methods

62 active youths (age = 11.7 ± 1.0 yrs.; females = 37, males = 25), were classified as pre-PHV, according to maturity status (Mirwald equation). Anthropometric measures (height, weight, body fat percentage, arm span, leg length, seated height) and physical performance tests (grip strength, vertical jump, prone bridge, modified sit and reach) were compared between girls and boys with the same maturity status.

## Results

Chronological age and anthropometric measures (height, weight, absolute arm span, absolute and relative seated height and absolute and relative leg length) were significantly higher in boys compared to girls (p=0.05), but body fat percentage was not different between girls and boys. Within the performance tests 1) boys scored significantly higher in absolute grip strength (R, p=0.00; L, p=0.00) absolute vertical jump height (p=0.008), and relative grip strength (kg/kg; mean ± SD)(left) (boys = 0.52 ± 0.12; girls = 0.45 ± 0.09; p=0.018), 2) girls scored significantly better than boys in absolute modified sit-and-reach test (p=0.05), and relative to stature (boys = 0.19 ± 0.03; girls = 0.24 ± 0.07; (cm/cm) p=0.01), and 3) there was no significant between-sex difference in relative vertical jump height (p=0.07) and absolute prone bridge time to exhaustion (p=0.29).

## Conclusions

In absolute performance measures there were significant differences between prepubertal boys and girls, and therefore, prescription of exercise and sport should be tailored accordingly.

# Abstract: O30: There is a high rate of medical encounters (1 in 50 runners) during the 90km “Comrades” ultra-distance race - a SAFER study in 84 117 ultramarathon runners

**Published:** 2019-01-01

**Authors:** Nicola Sewry, Jeremy Boulter, Martin Schwellnus

**Affiliations:** 1 Sport, Exercise Medicine and Lifestyle Institute (SEMLI), Faculty of Health Sciences, University of Pretoria, South Africa; 2 International Olympic Committee (IOC) Research Centre, South Africa; 3 Medical director, Comrades Marathon

## Introduction

The 90km “Comrades” ultramarathon is a popular annual event but there are limited data on medical encounters during this race. The purpose of this study was to determine the incidence rate of medical encounters (both illness and injuries) over multiple years during the 90km “Comrades” ultramarathon.

## Methods

A retrospective clinical audit during the “Comrades” ultramarathon (2014–2018) was conducted, and involved 84 117 race starters. Medical encounter (MEs) (moderate and serious life-threatening) data were collected over the years by medical doctors during and immediately after the event. Data were retrospectively coded using the 2019 international consensus definitions for MEs. Incidence rate (IR: per 1000 race starters) for all MEs, illness related MEs, injury-related MEs and organ-specific illness-related MEs were reported.

## Results

The overall IR for MEs over the five years was 21.0 (95%CI: 20.0–22.0) per 1000 starters (n=1 768). The IR for illness-related MEs (20.2; 95%CI: 19.3–21.2) was significantly higher than injury-related MEs (0.8; 95%CI: 0.6–1.0). The IR for serious life-threatening MEs was 1.9 (95%CI: 1.6–2.2) (1 in 526 starters). The highest IR, by affected organ systems for illness-related MEs, were: fluid and electrolyte (10.5; 95%CI: 9.8–11.2), central nervous system (4.0; 95%CI: 3.5–4.4), gastrointestinal (2.8; 95%CI: 2.4–3.1) and cardiovascular (1.2; 95%CI: 1.0–1.5).

## Conclusions

The IR (per 1000 starters) of MEs during the Comrades Marathon (21) is much higher compared to other endurance running events in South Africa (IR=5 in 21.1km Two Oceans and IR=13 in 56km Two Oceans). Risk factors associated with this high incidence require investigation so that interventions can be developed and implemented to improve safety of the participants’.

# Abstract: 031: Perceptions of training load and wellness monitoring of Stellenbosch University high performance student-athletes

**Published:** 2019-01-01

**Authors:** Sean Surmon, Lindsay Starling, Grant Van Velden, Wayne Derman, James C Brown

**Affiliations:** 1 Institute of Sport and Exercise Medicine, Division of Orthopaedics, Department of Surgical Sciences, Stellenbosch University, Cape Town, South Africa; 2 IOC Research Centre, South Africa; 3 Maties Sport, Stellenbosch University, Stellenbosch, South Africa

## Introduction

The effective monitoring of athletes can assist in optimising their performance. This monitoring is particularly important in university student-athletes who have academic stressors additional to their training. The Stellenbosch University (Maties) High Performance programme manages the top student-athletes and have implemented a training load and wellness monitoring system to assist with this. The aim of this study was to investigate student-athletes’ perceptions of this monitoring system and identify potential barriers to their adherence to the programme.

## Method

An internal survey was sent to all Maties High Performance Athletes (n=156) across six sporting codes. Four of this survey’s questions were based upon a study conducted in nine elite U.K. athletes and two additional questions were specific to the Maties High Performance context. Results were presented as frequencies on the original Likert scale.

## Results

Ninety-six % of athletes (n=149) answered this survey. Half (50%, n=74) of all athletes agreed that they received sufficient feedback from the data that they entered. Almost half (46%, n=69) agreed that sufficient action was taken by their Strength and Conditioning trainers when they indicated a meaningful change in their monitoring scores. Almost all (97%, n=144) athletes agreed that they responded honestly to training monitoring questions. About two-thirds (67%, n=100) of athletes agreed that training monitoring and feedback helped to optimise their training performances.

## Conclusion

Overall, the Maties High Performance student-athletes were substantially more positive about training monitoring than the elite UK athletes. This positivity bodes well for the Maties High Performance programme, but also highlights the importance of regular feedback to these student-athletes.

# Abstract: O32: Are training/running history, chronic diseases and medication use risk factors for injury prone runners? - a SAFER study in 75 421 race entrants

**Published:** 2019-01-01

**Authors:** Sonja Swanevelder, Nicola Sewry, Martin Schwellnus, Esme Jordaan

**Affiliations:** 1 Biostatistics Unit, South African Medical Research Council, South Africa; 2 Sport, Exercise Medicine and Lifestyle Institute (SEMLI), Faculty of Health Sciences, University of Pretoria, South Africa; 3 International Olympic Committee (IOC) Research Centre, South Africa; 4 Emeritus Professor of Sport and Exercise Medicine, Faculty of Health Sciences, University of Cape Town, South Africa; 5 Statistics and Population Studies Department, University of the Western Cape

## Introduction

Chronic injuries are common in recreational runners, and often interfere with training and competition. The aim of this study was to investigate risk factors associated with being “injury prone” in entrants for a mass community-based running event.

## Methods

Data from an online pre-race medical screening questionnaire (75 421 consenting entrants) were analysed, using a cross-sectional observational study design. Injury prone had 3 categories: 1) “none” (never injured) 2) “fairly” (less years injured than years entered), 3) “very” (always injured when entering). Risk factor categories (demographic info, running/training history, history of existing chronic diseases and medication use) were explored for being a “low” injury prone (LIP) runner by using an ordinal multinomial regression, reporting probabilities and odds ratios (OR) (with 95% CIs). Significance was at the 5% level.

## Results

Females, younger runners, 21.1 km (compared to 56 km) runners and runners with a higher body mass index (BMI) were associated with being a LIP runner. Running/training history, chronic diseases and medication risk factors were adjusted for gender, race distance and age category. Individual risk factors that were subsequently significantly associated (p<0.0001) with being a LIP runner were:1) certain running/training history variables, 2) Analgesic/anti-inflammatory (AAIM) medication use (OR=0.24), gastrointestinal disease (OR=0.39), symptoms for cardiovascular disease (CVD) (OR=0.41), haematological/immune disease (OR=0.47), nervous/psychiatric disease (OR=0.48), allergies (OR=0.49), respiratory disease (OR=0.50), kidney/bladder disease (OR=0.52), history of CVD (OR=0.52), prescribed chronic medication use (OR=0.56), risk factors for CVD (OR=0.57), metabolic disease (OR=0.65) and cancer (OR=0.85). Further analyses include a final multiple ordinal multinomial regression model.

## Conclusions

The most significant risk factors for runners not to be a LIP runner included AAIM medication, gastrointestinal disease, symptoms of CVD and an immune disease. These and some of the other risk factors should be further investigated for specific chronic injuries.

# Abstract: O33: Pacing characteristics of whole and part-game players in professional rugby union

**Published:** 2019-01-01

**Authors:** Jason Tee, Yoga Coopoo, Mike Lambert

**Affiliations:** 1 Department of Sport Studies, Faculty of Applied Sciences, Durban University of Technology, South Africa; 3 Department of Sport and Movement Studies, Faculty of Health Sciences, University of Johannesburg, South Africa; 4 Division of Exercise Science and Sports Medicine, Department of Human Biology, University of Cape Town, Cape Town, South Africa; 2 Carnegie Applied Rugby Research (CARR) centre, Institute for Sport, Physical Activity and Leisure, Leeds Beckett University, Leeds, United Kingdom

## Introduction

Contemporary theories on players’ intensity distribution in team sports suggest they regulate their outputs using pacing strategies. There is currently limited information on the effect of bout duration on pacing and movement patterns in rugby union match play. This study investigated the effect of different bout duration types (whole game, starter or finisher) on movement patterns of professional rugby union players within different position groups (forwards and backs).

## Methods

Global positioning system (GPS) and accelerometer data were collected from 100 professional match participations to determine temporal effects on movement patterns.

## Results

For forwards, finishers (players who entered the game as substitutes) demonstrated significantly greater high-speed running distance (% difference, ± 90%CI; magnitude based inference and effect size) (55, ±17%; *very likely* large) and acceleration frequency (78, ±59%; *very likely* large). Backs demonstrated no significant bout effects, but starters (players who started the game and were later substituted) tended (*p* = 0.07) to display greater high speed running distance than whole game players (+27, ±21%; ES = *likely* medium). Forwards displayed “slow-positive” pacing strategies regardless of bout type, while backs displayed “flat” pacing strategies.

## Conclusions

Forwards and backs adopt different pacing strategies regardless of bout type, with forwards demonstrating progressively greater performance decrements over the course of the match. These findings reflect differing physical demands, notably contact and running loads, of players in different positions.

# Abstract: O34: Effective working processes employed by a multidisciplinary team to reduce injury outcomes in a professional rugby league academy

**Published:** 2019-01-01

**Authors:** Jason Tee, Fieke Rongen

**Affiliations:** 1 Department of Sport Studies, Faculty of Applied Sciences, Durban University of Technology, South Africa; 2 Carnegie Applied Rugby Research (CARR) centre, Institute for Sport, Physical Activity and Leisure, Leeds Beckett University, Leeds, United Kingdom

## Introduction

In professional team sport managing injury risk depends on the effective collaboration of technical/tactical coaches, strength and conditioning coaches, sports medicine practitioners and sport scientists within a multidisciplinary team (MDT), yet to date no research has examined how these professionals might co-ordinate their efforts. The aim of this research is to examine the processes and interactions utilized by a demonstrably effective MDT.

## Methods

This research utilised a mixed methods design incorporating both quantitative and qualitative research methods. The efficacy of the MDT was established by quantifying the injury burden of the team across two seasons. Semi-structured interviews were conducted with all MDT members at the end of the second season, to determine each individual’s role within the injury prevention process and how these were co-ordinated between disciplines. Interviews were thematically analysed to provide insight into the approach adopted by the MDT.

## Results

The MDT achieved a 41% reduction in team injury burden during the second season in question (first season 3 450 vs. second season 1428 days/1 000 hours). Thematic analysis identified three key constructs that contributed to the MDT’s success; 1) shared values, 2) expertise and 3) the effective operationalization of underpinning values and expertise. Shared values expressed by the MDT members included doing what is best for players, rather than for the team, and shared responsibility for injury prevention. All MDT members were experts in their own fields but identified that they had to learn how to be effective within the environment and required skill in building interpersonal relationships. The operationalisation of these principals took place through iterative Plan-Do-Review cycles underpinned by sophisticated data collection and monitoring and the provision of time and resource to complete these operations.

## Conclusion

This research offers insights into “how” effective MDT’s work to achieve injury reduction, and the models and processes presented will inform practice for other MDT’s working in sport.

# Abstract: O35: Profile of Participants in the 19**^th^** World Transplant Games

**Published:** 2019-01-01

**Authors:** Johan van Heerden, Nicole Forman, Hayley Freimond, Sinothando Mbanjwa

**Affiliations:** Department of Biokinetics, Exercise and Leisure Science, University of KwaZulu-Natal, South Africa

## Introduction

The World Transplant Games (WTG) started in 1978 and are run every two years but research related to the event is limited. Accordingly, the purpose of this study was to analyse the profile of participants in the 19th World Transplant Games held in Durban, South Africa.

## Methods

The design comprised a questionnaire survey among participants (males and females) participating in the WTG. Statistical analysis included chi-square analysis and t-tests to distinguish statistical differences between sub-sets of responses with alpha set at p<0.05.

## Results

The mean age of respondents (n=60) was 39.51± 13.59 years with most falling into the category of 18–29 years (32.8%) and 50–59 years (29.5%) with the majority (p<0.05) being male (74%) compared to female (26%). The most common organ received (p<0.05), was a kidney (51.50%) followed by the heart (15.20%) and the liver (10.60%). The organ was generally received within the first 5 years of requiring a transplant (32.8%) and in most cases the time waited to receive the organ ranged between 0–6 months (35.1%) and between 7–24 months (35.1%). The majority (p<0.05) returned to physical activity within 6 months of receiving the organ (71%) and had been physically active for 9 years or more (73%; p<0.05). The majority (68%; p<0.05) were participating in the Games for the third time or more and the activity most commonly participated in was athletics (31.10%) followed by swimming (24.40%) and racquet sports (22.2%). Participants rated (scored out of 10) their mean health condition better (p<0.05) after transplant (8.3 ± 1.9) than before (4.6 ± 2.9) as well as their physical ability being greater (p<0.05) after transplant (8.2 ± 1.5) than before (4.9 ± 3.0).

## Conclusions

This study found that organ transplant improved participant’s perceived health and physical ability and demonstrates that organ transplant recipients can be rehabilitated successfully to lead normal lives and compete athletically.

# Abstract: O36: Presenting features of female collegiate sports related concussion: a descriptive analysis

**Published:** 2019-01-01

**Authors:** R. van Tonder, L. Kunorozva, P. L. Viviers, J.C. Brown, E. W. Derman

**Affiliations:** 1 Institute of Sport and Exercise Medicine, Division of Orthopaedics, Faculty of Health Sciences, Stellenbosch University, Stellenbosch, South Africa; 2 Campus Health Service, Stellenbosch University, Stellenbosch, South Africa; 3 IOC Research Centre, South Africa

## Background

Concussion is increasingly being recognised as an important sports related injury, especially in collision and contact sports. It constitutes a public health concern, given the large population at risk. The injury burden associated with concussion is high. Evidence suggests that females are at increased risk for SRC and have higher symptom burden before and after injury.

## Aims

The purpose of this study was to describe presenting features of female sports related concussion (SRC) at a collegiate campus-based health service.

## Methods

A retrospective cohort analysis of females presenting with SRC during the period 2012 – 2018 was performed. Concussion evaluation included the use of the Sports Concussion Assessment Tool (SCAT) versions 3 and / or 5. The SCAT questionnaire is focused inter alia on the self-reporting of symptom severity based on a scale of 0 – 6, where 0 indicates the absence of a symptom and 6 indicates the highest severity. There are 22 symptoms with a maximum possible severity score of 132.

## Results

43 (29%) females with SRC were identified, with a mean age of 23.2 (mean ± SD; 23.2 ± 3.1). Field hockey accounted for the largest number of SRCs, (21; 49%). The 3 most frequently reported symptoms were *headache* (85%), *feeling slowed down* (83%) and *pressure in head* (83%). The 3 symptoms with highest average severity score were *headache* (2.95), *feeling slowed down* (2.93) and *fatigue / low energy* (2.88). A history of prior concussion (n = 13) or anxiety and / or depression (n = 8) lead to higher average total severity scores of 51 (vs 39.8) and 62.9 (vs 38.6) respectively.

## Conclusion

Symptoms with the highest burden were *headache* and *feeling slowed down*. The average total symptom severity score appears to be higher in those with at least 1 previous concussion or a history of anxiety and / or depression.

# Abstract: O37: The relationship between physical activity and left carotid intima-media thickness among educators in South Africa: the SABPA study

**Published:** 2019-01-01

**Authors:** Tamrin Veldsman, Mariette Swanepoel, Andries Monyeki, Sanette Brits, Leone Malan

**Affiliations:** 1 Physical activity, Sport and Recreation Research focus area (PhASRec), Faculty of Health Sciences, North-West University, Potchefstroom, South Africa; 2 Hypertension in Africa Research Team (HART), School for Physiology, Nutrition and Consumer Sciences, North-West University (Potchefstroom campus)

## Introduction

Obesity and physical inactivity are associated with cardiovascular disease (CVD). The relationship between objectively measured physical activity (PA) and left carotid intimamedia thickness (L-CIMT) (carotid atherosclerosis marker) has sparsely been investigated. The aim of the study was to determine the relationship between PA status and L-CIMT among educators in South Africa.

## Methods

The cross-sectional study design was done on 216 urban-dwelling South African educators (χ̄ 49.67±8.44 years) from the Sympathetic Activity and Ambulatory Blood Pressure in Africans (SABPA) cohort study. A SonoSite Micromaxx ultrasound system measured L-CIMT and Actiheart determined consecutive seven days PA. Data were analysed by means of descriptive statistics, independent t-tests, chi-square and one way ANOVA. The relationship between activity energy expenditure (AEE) and L-CIMT according to PA categories was analysed using a Spearman and partial correlations.

## Results

More than a third of the participants were overweight and obese, and being more pronounced in the sedentary and light activity PA groups. Middle adulthood, showed a significantly higher mean (0.73±0.14 mm) (p>0.001) L-CIMT compared to young adulthood (0.64±0.16 mm). AEE was negatively associated with L-CIMT in the light intensity PA group (r=−0.02; p=0.84). A significant positive relationship was found between AEE and L-CIMT (r=0.69; p=0.001) in the moderate to high PA group. When partial correlation was calculated between AEE and L-CIMT (controlled for age, body mass index and waist circumference), respective significant positive relationships (*r* =0.48, p=0.05; *r*=0.99, p=0.001; *r*=0.53, p=0.02) in the overweight and obese middle adult age group, were observed.

## Conclusions

Participation in light intensity PA may be effective in the reduction of L-CIMT. The observed prevalence of overweight and obesity might have contributed to the positive relationship between AEE and L-CIMT. Given the health implication of these findings to CVD, urgent strategic physical activity intervention studies are recommended to improve the situation.

# Abstract: O38: Health promotion in individuals with spinal cord injuries: What is standing in the way of enhancing physical activity?

**Published:** 2019-01-01

**Authors:** Candace Vermaak, Suzanne Ferreira, Elmarie Terblanche

**Affiliations:** 1 Department of Sport Science, Faculty of Medicine and Health Sciences, Stellenbosch University; 2 Institute of Sport and Exercise Medicine, Faculty of Medicine and Health Sciences, Stellenbosch University

## Introduction

Barriers to physical activity are universal, however, in a developing country and in individuals with a spinal cord injury (IwSCI) these barriers are often exacerbated and multiple. Whereas, facilitators are crucial in promoting and sustaining physical activity (PA) behaviour. Thus, the aim of the study was to determine the barriers and facilitators to PA for IwSCI within the Cape metropole.

## Method

A mixed-method research design was used. A self-developed questionnaire was used to gather the information regarding the barriers and facilitators to PA. The barriers and facilitators were themed according to the theory of planned behaviour into personal, social, environmental and program/policy. Fifty seven IwSCI (men: n =49; women: n = 8) completed the questionnaire.

## Results

Environmental barriers were most prevalent (40%) and included limited facilities and transport, as well as difficulties related to the weather. Program barriers (33%) included lack of trained volunteers and appropriate programs, while personal barriers (20%) included injury level and secondary conditions. The social barriers accounted for 7% and included lack of information received from therapists regarding physical activity. The facilitators identified included enjoyment, a desire to be physically active (personal 44%), family support, support from peers with disabilities (social 26%), accessibility and the facility being a safe location (environmental 17%), skilled staff and better quality programs (program/policy 13%).

## Conclusion

It is evident that IwSCI face various obstacles in being physically active, which ultimately obstructs the journey to health and wellness. If health and wellness is not achieved, quality of life is affected and it can lead to many other co-morbidities and even early mortality. The findings demonstrates the importance of identifying and the removal of such barriers to promote and foster effective interventions for an active and healthy lifestyle for IwSCI.

# Abstract: O39: Acute injury medical encounters (MEs) are 3.9 times more common than illness MEs in mass community-based trail running events: SAFER study in 2428 race starters

**Published:** 2019-01-01

**Authors:** Carel Viljoen, Nicola Sewry, Martin Schwellnus, Dina C (Christa) Janse van Rensburg, Sonja Swanevelder, Esme Jordaan

**Affiliations:** 1 Sport, Exercise Medicine and Lifestyle Institute (SEMLI) and Department of Physiotherapy, Faculty of Health Sciences, University of Pretoria, South Africa; 2 Sport, Exercise Medicine and Lifestyle Institute (SEMLI), Faculty of Health Sciences, University of Pretoria, South Africa; 3 International Olympic Committee (IOC) Research Centre, South Africa; 4 Sport, Exercise Medicine and Lifestyle Institute (SEMLI) and Section Sports Medicine, Faculty of Health Sciences, University of Pretoria, South Africa; 5 Emeritus Professor of Sport and Exercise Medicine, Faculty of Health Sciences, University of Cape Town, South Africa; 6 Biostatistics Unit, South African Medical Research Council; 7 Statistics and Population Studies Department, University of the Western Cape, South Africa

## Introduction

Trail running is characterized by “off-road” running on uneven and varying running surfaces perhaps predisposing runners to more acute injuries during races compared with road running. The aim of this study was to describe the epidemiology of race-day injury-related vs. illness-related medical encounters (MEs) during a mass community-based trail running event over 3 years.

## Methods

In this retrospective clinical audit in 2428 race starters during the 2012–2014 Two Oceans Trail Runs (10km and 22km), injury-and illness-related MEs, defined as at least of moderate severity (2019 consensus statement), were recorded by race medical staff. Crude incidence rate (IR per 1000 starters; 95%CI’s) of MEs (all, injury-related and illness-related) and the IR of specific injuries (anatomical region) and illnesses (organ systems affected) were reported.

## Results

44 MEs were reported in 2428 race starters with an overall IR of 18.1 (12.8–23.5). The IR for all injury-related MEs was significantly higher than all illness-related MEs [14.4 (9.6–19.2) vs. 3.7 (95%CI;1.3–6.1)]. The highest injury-related IR (IR;95%CI) in specific anatomical regions was the knee (4.5;1.9–7.2), followed by the ankle (2.1;0.3–3.9). Lacerations/abrasions (IR=8.2,95%CI;4.6–11.8), and ligament sprains (IR=2.1;0.3–3.9) were the most common tissue type injured. All ligament sprains were in the ankle. Exercise associated postural hypotension (IR=1.6;0.0–3.3) was the most common illness-related ME.

## Conclusion

At the Two Oceans trail running races, acute injury-related medical encounters (MEs) are 3.9 times more common than illness-related MEs. In addition, the IR of overall MEs in the trail running races are higher than in the 21.1km (IR=5.1) and 56km (IR=13.0) road running events. Finally, there is a particularly high IR of acute ankle ligament sprains in trail running. These findings are important for trail running race organizers and supporting medical teams to prepare for race day, and to develop prevention programs for MEs.

# Abstract: O40: Over 14% trail runners report an overuse injury in a 12-month period, mostly affecting the knee and anterior thigh: A SAFER study in 2824 race entrants

**Published:** 2019-01-01

**Authors:** Carel Viljoen, Nicola Sewry, Martin Schwellnus, Dina C (Christa) Janse van Rensburg, Sonja Swanevelder, Esme Jordaan

**Affiliations:** 1 Sport, Exercise Medicine and Lifestyle Institute (SEMLI) and Department of Physiotherapy, Faculty of Health Sciences, University of Pretoria, South Africa; 2 Sport, Exercise Medicine and Lifestyle Institute (SEMLI), Faculty of Health Sciences, University of Pretoria, South Africa; 3 International Olympic Committee (IOC) Research Centre, South Africa; 4 Sport, Exercise Medicine and Lifestyle Institute (SEMLI) and Section Sports Medicine, Faculty of Health Sciences, University of Pretoria, South Africa; 5 Emeritus Professor of Sport and Exercise Medicine, Faculty of Health Sciences, University of Cape Town, South Africa; 6 Biostatistics Unit, South African Medical Research Council; 7 Statistics and Population Studies Department, University of the Western Cape, South Afria

## Introduction

Trail running differs from road running because of uneven and varying running surfaces, larger changes in altitude and large elevation gains/losses. Injury patterns in trail running may differ from road running, but this has not been well studied. The aim was to determine the epidemiology (annual incidence) and nature of self-reported overuse/chronic injuries among trail runners (10km and 22km) participating in a mass community-based trail running events over a 4-year period.

## Methods

A retrospective descriptive cross-sectional study was performed using data collected from a pre-race medical screening questionnaire completed by 2824 consenting entrants (79.6% of all participants) during the 10km and 22km Two Oceans Trail Runs (2012–2015). Self-reported injury history, in the previous 12 months included data on anatomical site, tissue type, specific diagnosis and injury severity grade. Main outcome variables were retrospective annual incidence (%runners:95% CI) and frequency (%) of injury characteristics by anatomical site, tissue type, specific running injury, and injury severity grade (1–4).

## Results

406 injuries in total were reported (12 participants reporting 2 injuries) with a retrospective annual incidence of 14.4% (95% CI;13.0–15.8). Most commonly injured anatomical site were the knee (25.2%), anterior thigh (10.0%), shin/lower leg (8.4%) and ankle (8.4%). Common injuries by tissue type were muscle (30.3%) and tendon (28.9%). The most common specific running injuries were Iliotibial band syndrome (ITBS) (20.0%), Achilles tendon (8.6%) and hamstring injuries (7.9%). Over 50% of the injuries were of Grades 3 and 4 severity.

## Conclusion

14.4% trail runners report an injury in a 12-month period, most commonly affecting the knee and anterior thigh. Over 50% of injuries were severe enough to affect training or racing. Risk factors associated with injuries in trail runners need to be determined, in order to design and implement prevention strategies.

# Abstract: O41: Exercise Associated Muscle Cramp (EAMC) is almost twice as common among 21.1km compared to 56km runners – A SAFER study in 76654 runners

**Published:** 2019-01-01

**Authors:** Izaan De Jager, Martin Schwellnus, Carel Viljoen, Elzette Korkie, Sonja Swanevelder, Esme Jordaan

**Affiliations:** 1 Sport, Exercise Medicine and Lifestyle Institute (SEMLI) and Department of Physiotherapy, Faculty of Health Sciences, University of Pretoria, South Africa; 2 Sport, Exercise Medicine and Lifestyle Institute (SEMLI), Faculty of Health Sciences, University of Pretoria, South Africa; 3 International Olympic Committee (IOC) Research Centre, South Africa; 4 Emeritus Professor of Sport and Exercise Medicine, Faculty of Health Sciences, University of Cape Town, South Africa; 5 Biostatistics Unit, South African Medical Research Council; 6 Statistics and Population Studies Department, University of the Western Cape, South Africa

## Introduction

Exercise associated muscle cramps (EAMC) among distance runners has not been well studied. Therefore, this study aimed to describe the epidemiology, clinical characteristics and severity of EAMC in distance runners, and compare these characteristics between the 21.1km and 56km runners.

## Methods

76654 consenting runners completed an online pre-race medical screening questionnaire as part of the 2012–2015 Two Oceans race entry process (21.1km=47069; 56km=29585). The main outcome variables (adjusted for age and gender) are: lifetime prevalence, clinical characteristics (main muscle groups affected, timing of occurrence during a race, EAMC severity, complex forms of EAMC, and treatment of EAMC) in all runners and sub-groups (21.1km vs. 56km) (%; 95%CI).

## Results

The lifetime prevalence of EAMC of all runners was 12.8% (12.6–13.1), and this was significantly higher in 21.1km (16.0%; 15.5–16.5) vs. 56km runners (8.8%; 8.5–9.1) (p=0.0001). The main anatomical regions affected by EAMC in all entrants and in 21.1km and 56km runners were the calf (All=53.1%; 52.1–54.2), hamstrings (All=20.9%; 20.0–21.7) and quadriceps muscles (All=16.3%; 15.5–17.1). The majority of EAMC occurred in the fourth quarter of a race (All=46.4%; 45.4–47.5, 56km=35.1%; 33.6–36.6, 21.1km=47.2%; 45.6–48.9) followed by after the race (All=22.3%; 21.4–23.2, 56km = 29.5%; 28.1–31.1: 21.1km = 18.6%; 17.5–19.8). The severity of EAMC was classified by all runners as mild (65.2%; 64.2–66.2) followed by moderate (29.9%; 29.0–30.9). Complex severe EAMC (6.3%; 5.8–6.8), whole body cramping EAMC (3.7%; 3.4–4.2) and EAMC associated with dark urine (2.6%; 2.3–3.0) were reported. All runners indicated that stretching relieved an acute cramp most successfully (70.2%; 69.2–71.1), followed by rest (37.9%; 36.9–38.9) and massage (37.9%; 37.0–39.0).

## Conclusion

About 12.8% of recreational runners report ever suffering from EAMC and this was significantly higher in 21.1km sub-group (16.0%), compared to 56km runners (8.8%). Most EAMC in distance runners occurs in the calf and thigh muscles, presenting later 4^th^ quarter and post-race.

# Abstract: O42: A history of multiple chronic diseases is associated with Achilles tendon injuries in runners: A cross-sectional SAFER study in 76654 runners

**Published:** 2019-01-01

**Authors:** Jonah Young, Martin Schwellnus, Kim Nolte, Sonja Swanevelder, Esme Jordaan, Paola Wood

**Affiliations:** 1 Sport, Exercise Medicine and Lifestyle Institute (SEMLI) and Division of Biokinetics and Sports Science, Department of Physiology, Faculty of Health Sciences, University of Pretoria, South Africa; 2 Sport, Exercise Medicine and Lifestyle Institute (SEMLI), Faculty of Health Sciences, University of Pretoria, South Africa; 3 International Olympic Committee (IOC) Research Centre, South Africa; 4 Emeritus Professor of Sport and Exercise Medicine, Faculty of Health Sciences, University of Cape Town, South Africa; 5 Biostatistics Unit, South African Medical Research Council; 6 Statistics and Population Studies Department, University of the Western Cape

**Keywords:** Achilles tendon, overuse running injuries, chronic disease, medication use, intrinsic risk factors

## Purpose

Achilles tendon injuries (ATI) in runners is associated with multiple risk factors, with recent data showing a possible association between ATI and chronic metabolic disease. The aim of this study was to determine whether chronic disease, in addition to previously reported risk factors, is associated with ATI in the Two Oceans Marathon runners (2012–2015).

## Methods

Online pre-race medical screening questionnaire data from 76654 consenting race entrants (71.8% of all entrants) were analysed, using a cross-sectional observational study design. A total of 739 (1.2%) entrants reported an ATI. The following categories of risk factors associated with ATI were explored using multi-variate analyses, reporting prevalence ratios (PR): race distance (21.1 km vs. 56 km), runner demographics, training/racing history, history of existing chronic disease, and medication use.

## Results

When comparing race entrants who reported an ATI to those who did not older age (>31yrs)(*p<0.0001*), male sex (PR=1.76; *p<0.0001*), and longer race distance (56km vs. 21.1km)(PR=2.06; *p<0.0001*) were associated with a higher prevalence of ATI. Independent risk factors associated with ATI (adjusted PR for race distance, sex and age) were: increased years of recreational running (*p<0.0001*), risk factors for cardiovascular disease (CVD) (PR =1.77; *p*<0.0001), history of chronic gastrointestinal tract (GIT) disease (PR =2.06; *p=*0.0155), respiratory disease (PR =1.66; *p*<0.0001), nervous system/psychiatric disease (PR =1.56; *p*<0.0001), allergies (PR =1.89; *p*<0.0001) and history of use of analgesic/anti-inflammatory medication use (PR =4.83; *p*<0.0001).

## Conclusion

The main findings of this study apart from age, race distance and male sex are that novel independent risk factors associated with ATI include years of being a recreational runner, risk factors for CVD, history of respiratory or GIT or nervous system/psychiatric disease, allergy and analgesic/anti-inflammatory medication. These should be considered when implementing prevention programs and planning effective race medical support systems whilst future studies should focus on a establishing a causal relationship.

# Abstract: C01: Sudden onset lateral foot pain in a tri-athlete

**Published:** 2019-01-01

**Authors:** Emmari Carstens, Susan Crumpton, Dr PL Viviers

**Affiliations:** 1 Institute of Sport and Exercise Medicine, Stellenbosch University, South Africa; 2 IOC Research Centre, Cape Town, South Africa; 3 Campus Health Services, Stellenbosch University, South Africa

## History

A healthy 22-year-old male presented to the Sports Clinic complaining of lateral left sided foot pain. He was in training for a Half Iron-man event and had countless failed attempts at conservative management over the past 4 months. The pain started gradually within 2 running sessions with no clear mechanism of injury. Initially he consulted his family physician who suspected a stress fracture and referred him for x-rays, which came back clear. He was management non-weightbearing in a moonboot for 3 weeks. After 3 weeks, he was still symptomatic and consulted a foot and ankle specialist. His findings were tenderness of the peroneus longus tendon and groove. He was referred for a MRI that also came back clear. His advice was to see a Biokineticist for rehabilitation. Static and dynamic loading patterns were done by a Podiatrist thereafter, who found cavo-varus feet and calcaneus hypermobility. He advised orthotics to help with load distribution. He continued managing his foot with orthotics and rehabilitation, which focused on improving foot contact and loading during running and general hip muscle recruitment. After 4 weeks of rehabilitation, he started with gradual return to running and again experienced pain after 1km. He presented to the Sports Clinic, were findings were no swelling or bruising, hypomobility of SIJ with hip movements on the left and severe gluteus medius spasm. The hypothesis was a glut medius triggerpoint referring laterally into the foot and he was referred to physiotherapy. Previous relevant history includes episodes of recurring ITB syndrome in the past 3 years and one episode of patella tendinopathy and patella femoral pain syndrome, all on the left side. Normal medical and family history and didn’t use any medication to manage his symptoms.

## Physical findings

Generally healthy and active individual With presentation at the physiotherapy clinic, he had no pain with any functional testing of the lower limb and was able to jog and sprint pain free for about 50m. With hip flexion in standing, the SIJ on the left was not moving inferiorly. With palpation of the lumbar area he had tightness of quadratus lumborum and stiffness of levels L3-S1 centrally and unilaterally to the spine, left more than right.

At the foot, had tenderness of the plantar fascia and hypermobility of the calcaneus on the left compared to the right. He also had mid-foot stiffness, specifically with pronation and inversion of the forefoot compared to the right.

## Differential Diagnosis

Functional foot pain due to loading on the lateral part of the foot

- Biomechanical discrepency secondary to soft tissue tightness
- Midfoot joint stiffness which doesn’t allow for normal biomechanical movement when running

## Hypothesis

Biomechanical discrepancy due to soft tissue tightness of the gluteus medius and foot immobility leading to increased load on the lateral side of the foot.

## Management

- Specific soft tissue mobilisations of the quadratus lumborum and gluteus medius
- Mobilisation of the lower back T12-S1 centrally and unilaterally
- Dry needling of the gluteus medius
- Midfoot and forefoot mobilisation
- Strapping to support calcaneus during running

## Outcome

After 2 physiotherapy sessions, patient was able to run more then 2km painfree and after the 3^rd^ session, was able to complete the Half Iron man 21 km symptom free. He returned to previous level of activity after his 3^rd^ session.

# Abstract: C02: A multistructural knee injury in a female field hockey player

**Published:** 2019-01-01

**Authors:** Tanya Green, Susan Crumpton, Dr PL Viviers, Prof Wayne Derman

**Affiliations:** 1 Institute of Sport and Exercise Medicine, Stellenbosch University, Cape Town, South Africa; 2 IOC Research Centre, Cape Town, South Africa; 3 Campus Health Services, Stellenbosch University, Cape Town, South Africa

## History

A 20-year-old female field hockey player presented with a painful knee post sustaining a multi-structural acute right knee injury after colliding with an opposition. The mechanism was a twist with hyperextension, no pop or tearing sound reported. She experienced immediate pain and was unable to bear weight, which forced her to discontinue playing. Immediate field-side management included ice, compression and immobilisation. The tournament physician examined her and made the diagnosis of lateral collateral ligament sprain (LCL) with lateral posterior meniscus injury, and referred her for further investigation when she returns home. On the first day after injury, the injured knee was swollen, painful and she was unable to straighten the leg. Upon returning home a sports physician was consulted who requested an MRI investigation and orthopaedic surgeon’s opinion. The clinical findings of the orthopaedics’ examination noted a small effusion, limited range of motion and tested positive for partial posterior collateral ligament (PCL) instability. The MRI confirmed a partial thickness tear of the PCL, bone contusion or impaction fracture of the anterolateral tibial condyles, associated meniscal contusion or flap tear of the anterior horn of the lateral meniscus. Conservative management was opted, which included immobilization in a brace and referred for physiotherapy. Medical and family history were normal and the athlete was not on chronic medication or using any supplements. Previous sport related injuries includes right hamstring strain (June 2017), concussion (August 2017) and left sided lumbar-sacral back pain (earlier in the year) which all resolved.

## Physical Findings

Generally healthy and physically active female field hockey player. Initially presenting at the physiotherapy clinic partial weight bearing (PWB) on crutches and immobilised in a PCL brace limited to 90° of knee flexion, with a moderate effusion. Her passive range of motion was limited to 0–80° at this point she experienced pain and stiffness with both movements. Pain ranging from 5–8/10 at worst on Numeric Pain Rating Scale (NPRS). Quadriceps lag of −15° due to stiffness and pain inhibition. Pain and tenderness on palpation anterolateral over the lateral joint line, distal insertion ITB and anterior knee soft tissue structures (due to the effusion), overall tightness of quadriceps and hamstrings.

## Differential Diagnosis/Hypothesis

Lateral collateral ligament injury; anterior cruciate ligament injury; posterior cruciate ligament injury; lateral meniscus injury/ contusion; posterior lateral corner injury, bone contusion of femoral or tibial condyles, and associated lateral and posterior soft tissue injuries.

## Final/ Working Diagnosis

Partial PCL tear, bone contusion of anterolateral tibial condyle with a small impaction fracture and contusion of the anterior horn of the lateral meniscus.

## Management and Treatment Outcomes

Immobilization partial weight bearing on crutches and in PCL brace for 4 weeks guided by orthopaedic surgeon only to mobilise for knee flexion up to 90° with anterior tibial translation at all times

- Physical therapy: manual therapy including soft tissue and joint mobilisation, electrotherapy modalities, taping, activation and strengthening of vastus medialis oblique, quadriceps and gluteal complex.
- Gradual mobilisation to full weight bearing
- Currently busy with rehabilitation under supervision of a biokineticist, focussing on sport-specific strengthening and proprioceptive training
- Aim to full integration to training and return to sport after 4 months

# Abstract: C03: An unexpected cause of leg pain in a 42 year old endurance runner

**Published:** 2019-01-01

**Authors:** Melissa Janse van Vuren

**Affiliations:** Institute of Sport and Exercise Medicine, Stellenbosch University, South Africa, IOC Research Centre South Africa

## History

A 42 year-old long-distance runner presented to the sports medicine clinic two weeks after having successfully completed the Two Oceans marathon complaining of left calf pain. The onset of the pain occurred the day after the ultra-marathon but within a short period the pain was present after walking short distances. The pain was claudicant in nature with accompanying foot paraesthesia and leg weakness. Rest pain was absent.

Other than a history of treatment naive hypercholesterolaemia and previous kidney stones, the patient was fit, and well, a non-smoker with some symptoms of atopy but no other symptoms of claudication or angina. There was no other relevant medical or family history.

## Physical examination

- A normal general examination and vital signs.
- There was no obvious swelling, discoloration or evidence of tissue loss of the lower limbs.
- The left limb was slightly cooler to touch with a prolonged capillary refill time.
- Further examination of his pulses, his upper limb pulses were symmetrical and equal but on examination of his lower limbs his left femoral pulse was slightly diminished, with a soft bruit. The left popliteal and dorsalis pedis pulses were absent.
- He was unable to run, with a claudication distance of 100 meter.
- No left foot pulses felt post exercise or heard on office Doppler examination.

## Differential results

Clinical evaluation strongly indicated a vascular cause of this presentation and the patient was urgently referred to a vascular surgeon for further evaluation.

1. Clotting factor pathology
2. Arterial dissection
3. Atherosclerotic disease 4. Vasculitis

## Test Results

Duplex Doppler was performed but slightly obscured by bowel gas, yet aorta and proximal common iliac seemed to be patent. The distal common iliac and proximal external iliac could not be visualized, but the distal external iliac and common femoral vessels were patent with biphasic flow.

Angiogram performed of the external iliac artery, demonstrated a string-like appearance pathognomonic of fibromuscular dysplasia. A full visceral angiogram revealed no involvement of the visceral vessels. The distal angiogram was normal.

## Working Diagnosis

Fibromuscular dysplasia of the external iliac artery

## Treatment and outcomes

1. Arteriotomy and thrombectomy was performed on the superficial femoral artery and the long saphenous vein was harvested and used as a venous patch.
2. 4 hours post-op review revealed a well perfused limb with good dorsalis pedis pulse. The patient was placed on Xarelto for anticoagulation.
3. The patient was treated with initial rest with gentle mobilization and attention to maintaining range of motion.
4. A return to sport programme was then successfully instituted.

# Abstract: C04: Persistent pain following ankle sprain: an unusual presentation of an osteoid osteoma

**Published:** 2019-01-01

**Authors:** Dr WM Sadie, Prof EW Derman

**Affiliations:** Institute of Sport and Exercise Medicine, Stellenbosch University, South Africa; IOC Research Centre, South Africa

## History

A 19-year-old female hockey athlete presented with an 18-month history of right sided ankle pain following a suspected ankle sprain whilst playing hockey. She was referred to the clinic by a physiotherapist who noted lateral ankle swelling and pain out of relation to the injury. Furthermore, she was not responding to physiotherapy treatment.

She had also been to consult an orthopaedic surgeon who had assessed her ankle as having a peroneus brevis tendinopathy, and an anomalous insertion of the peroneus tendon. These abnormalities had been reported at point of care ultrasound scanning. This condition was managed by corticosteroid injection which temporarily relieved the pain. After the pain returned a high volume injection was given in the painful area without any effect.

Further specific history, general medical and surgical history was not contributory; except for night pain, and response of pain to NSAIDS, which she had been ingesting for the previous year. Previous orthopaedic history revealed some previous lower back pain relieved by physiotherapy.

## Physical examination

Physical examination revealed some discomfort of the right ankle when bearing weight and jumping. There was visible swelling over the medial malleolus. Range of motion was normal. There was extreme tenderness of the lateral malleolus and pitting oedema present on palpation.

## Differential diagnosis

Complicated ankle sprain with missed fracture

Fracture with mal/non-union, or bone stress injury

Occult osteochondral lesion

Peroneus tendon injury

Complex regional pain syndrome

Osteoid osteoma

## Special investigations

Blood labs – within normal range

X-Ray of the ankle revealed cortical thickening with irregular margins of the distal fibular and associated soft tissue swelling. No nidus was identified on X-ray.

MRI - Routine sagittal, axial and coronal studies with additional fat saturation sequences. 1.5 Tesla body scanner and CT scan were performed. These investigations revealed extensive bone marrow edema of the distal fibula with associated nidus. There was also significant surrounding sclerosis as well as periosteal new bone formation seen with extensive surrounding soft tissue edema. The features were compatible with an osteoid osteoma.

## Final Diagnosis

Osteoid osteoma of the right distal fibula.

## Treatment and Outcomes

The patient was referred for radio-ablation which was successfully performed and a return to sport programme followed. The patient continues to recover well. Osteoid osteoma is a rare cause of ankle pain. The purpose of this case study is to highlight to healthcare providers to be aware of rare causes of chronic persistent pain in the athlete who does not respond to the expected course of recovery.

# Abstract: C05: Case report: a complicated shoulder dislocation in a collegiate rugby player

**Published:** 2019-01-01

**Authors:** Dr Craig Thompson, Dr PL Viviers, Prof Wayne Derman

**Affiliations:** 1 Campus Health Service, Stellenbosch University, Cape Town, South Africa; 2 Institute of Sport and Exercise Medicine, Stellenbosch University, Cape Town, South Africa; 3 IOC Research Centre, Cape Town, South Africa

## History

A 23-year-old male collegiate rugby player injured his left shoulder whilst playing in a rugby union match. He was tackled and landed on the pitch on an overstretched left arm (shoulder abducted and flexed at point of contact with the pitch), whilst carrying the ball at speed. He had had no history of previous shoulder injuries.

## Physical examination

Clinical signs with the on-field medical assessment showed that the player supported his left arm in an abducted and a forward flexed position. An empty defect was palpable below the left acromion and the humeral head was palpable in the left anterior axillary area. Neurovascular status in the left arm was intact. The player was removed permanently from the field of play. Evaluation in the medical room by the match day doctor confirmed an anterior left glenohumeral dislocation, which was relocated without effort. Post-reduction neuro-vascular status of the injured limb remained intact. Whilst in the medical room, a spontaneous recurring left shoulder dislocation occurred, which was again reduced without effort and with no change in the limb neurovascular status post-reduction.

## Differential diagnoses

1. Isolated anterior glenohumeral dislocation
2. Humeral or clavicular fracture, with shoulder dislocation
3. Rotator cuff or other ligamentous injury, with shoulder dislocation
4. Bony Bankart lesion, with shoulder dislocation
5. Hill-Sachs lesion, with shoulder dislocation.

Test results:

### X-ray (left shoulder)

Successful reduction of humeral head.
No other bony abnormalities (including Hill-Sachs or Bony Bankart lesions) could be demonstrated.
Non-specific mineralization was noted in relation to the supraspinatus tendon, a possible avulsion fracture.
No humeral fractures were noted.

### MRI (left shoulder)

Humeral avulsion of the gleno-humeral ligament (HAGL injury), with retraction was demonstrated.
Full-thickness tear of supraspinatus tendon, with minimal retraction.
Articular surface tear of subscapularis tendon.
Gleno-humeral joint effusion.
SLAP lesion (biceps anchor).
Muscle oedema (subscapularis and infraspinatus muscles)

## Working diagnosis

Acute antero-inferior left shoulder dislocation, complicated by HAGL lesion and multiple rotator cuff tears.

## Treatment and outcomes

1. Referral – orthopaedic surgeon for surgical management through an open procedure.
2. Post-operative physiotherapy rehabilitation:
  - Mobilization and range of motion - complete mobility achieved after 14 weeks
  - Strengthening - commenced after full mobility
3. Final outcome – the athlete is still busy with a shoulder strengthening rehabilitation program. He remains asymptomatic and functional but does not yet meet criteria for return-to-play.

# Abstract: P01: An investigation of the knowledge of South African high school rugby coaches on concussion and the return to play protocol

**Published:** 2019-01-01

**Authors:** NC Abel, CC Grant, DC Janse van Rensburg

**Affiliations:** Section Sport Medicine, Faculty of Health Sciences, University of Pretoria, South Africa

## Introduction

Concussion is a complex pathophysiological process affecting the brain. The coach plays a pivotal role in the management of the concussed player. Assessing the knowledge of the high school rugby coaches in South-Africa may enable future education on the subject to focus on areas where knowledge is lacking.

## Methods

A cross-sectional, descriptive study design was used with the aid of a validated questionnaire. First team rugby coaches of South African high schools were asked to complete questionnaire. Relationships between total scores for different demographic groupings were established using non-parametric techniques due to the presence of small and uneven sample sizes of the groups.

## Results

Symptom recognition score of 78% and general concussion knowledge score of 80% compare well with international study results. Maddock question knowledge score of 26% and return-to-play knowledge score of 62% were not adequate. Educational programs (Boksmart) is the most popular source of knowledge followed by healthcare providers. choice. Boksmart accredited coaches did significantly better than the non–accredited coaches in the total score knowledge score (p=0.041). Large category school coaches scored better in general concussion knowledge compared to small school coaches (p=0.0084). A significant difference was observed between the different coach age groups with the 35–44 year group indicating the best scores. Less than 3 years in coaching and the least qualified coaches scored worse than the other groups.

## Conclusions

South African high school rugby coaches do not have sufficient knowledge to manage the return-to-play of concussed players adequately. Boksmart accreditation, the size of the school and the age group of the coaches were identified as predictors for superior knowledge. Coaches at small schools were identified as a possible group with inferior knowledge of concussion.

# Abstract: P02: Changes in heart rate variability while exercising in humid and high temperatures

**Published:** 2019-01-01

**Authors:** M Bester, CC Grant, P Becker, DC Janse van Rensburg

**Affiliations:** 1 Section Sports Medicine, Faculty of Health Sciences, University of Pretoria, South Africa; 2 Faculty of Health Sciences, University of Pretoria, South Africa

## Introduction

During competition in hot environments, endurance athletes often exercise/compete at intensities that stress their cardiovascular system. The primary aim of the study was to quantify the effect of increased core temperature (while exercising in a hot and humid environment) on autonomic cardiac control and to determine if heart rate variability (HRV) indicators may be indicative of a rise in core temperature.

## Methods

The study was conducted in a climatic chamber where healthy male volunteers (n=14) exercised in a hot and humid environment for 120 minutes while core temperatures increased from normal, 37°C, to 39°C. RR interval data was continuously sampled for 120 minutes and compared with recorded baseline values (sampled during exercise in the same environment at normal core temperature: 37°C).

## Results

Increases in core temperature were accompanied with a rise in HR (84 bpm to 124 bpm, p=0.0034) which was largely due to vagal withdrawal and an overall decrease in all HRV indicator values: a)time domain indicators (Mean RR: 663.64 to 502.86, p=0.0034; STDRR: 26.45 to 18.46, p=0.031; RMSSD: 22.83 to 11.07, p=0.03; pNN50: 4.63 to 1.12, p=0.02), b) frequency domain indicators (LFms^2^: 437.86 to 141.50, p=0.011; HFms^2^: 197.29 to 45.89, p=0.0085) and c) non-linear HRV indicators (SD1: 16.34 to 8.09, p=0.036; SD2: 64.03 to 22.44, p=0.0099). However, indicators of autonomic balance all showed non-significant changes: LFNU: p=0.504; HFNU: p=0.517and LF/HF: p=0.395. Indicators of complexity and self-similarity gave heterogeneous results (SampEn and DFA). From the exploratory regression analyses, it was observed that MNRR (R_2=0.463), MNHR (R_2=0.59) and DFAα1 (R_2=0.350) show the most promise with regards to HRV indicators that could act as predictive indicators of elevated core temperature.

## Conclusion

Quantification of HRV indicators in young and healthy athletes indicated that increases in core temperature, during exercise, decreased all variability measures while maintaining autonomic balance. Furthermore, HRV indicators such as MNRR, MNHR and DFAα1 may have potential to indicate a rise in core temperature as there is a clear association between core temperature and these HRV indicators.

# Abstract: P03: The influence of match characteristics on post-match heart rate variability of male badminton players

**Published:** 2019-01-01

**Authors:** H Pienaar, B Coetzee, CA Bisschoff

**Affiliations:** Physical activity, Sport and Recreation Research Focus Area, Faculty of Health Sciences, North-West University, Potchefstroom Campus, Potchefstroom, South Africa

## Introduction

The purpose of this study was to investigate the influence of match characteristics on the post-match heart rate variability (HRV) of male badminton players.

## Methods

22 male, African, singles badminton players (age: 23.3 ± 3.9 years) participated in the study. During 46 national and international matches players were fitted with a Fix Polar Heart Rate Transmitter Belt and a MinimaxX Global Positional System (GPS) unit to record variations in beat to beat (R-R) intervals for 60 seconds directly after play had stopped (post-match) and court movements during matches.

## Results

Moderate significant, inverse Spearman’s rank correlations (p ≤ 0.1) were found between peak very-low frequency power (VLFP), distance covered, and time spent in the low intensity (LIZ) (r_s_ = −0.42; r_s_ = −0.33) and medium (r_s_ = −0.41; r_s_ = −0.41) intensity (MIZ) zones, respectively as well as between peak low frequency power (LFP) and time spent in the high intensity zone (HIZ) during matches (r_s_ = −0.33). Peak high-frequency power (HFP) showed a moderate significant inverse correlation with absolute and relative heart rate (HR) in the LIZ (r_s_ = −0.44) and a moderate significant positive correlation with the absolute and relative HR in the HIZ (r_s_ = 0.40). Lastly, moderate significant inverse correlations were also observed between LIZ and MIZ total player load and VLFP, respectively (r_s_ = −0.41; r_s_ = −0.38).

## Conclusions

Therefore, match characteristics can significantly influence the post-match HRV of male badminton players, although the direction and nature of the influence are not in line with what previous research indicate.

# Abstract: P04: 66% of 1**^st^** year university medical students report low habitual physical activity levels

**Published:** 2019-01-01

**Authors:** Jill Borresen, Martin Schwellnus, Paola Wood, Kim Nolte, Tanya Camacho, Martin Bac, Debashis Basu, Gerda Gericke, Amanda Talma, Marianne Schwellnus

**Affiliations:** 1 Sport, Exercise Medicine and Lifestyle Institute (SEMLI), University of Pretoria, South Africa; 2 Division of Biokinetics and Sport Science, University of Pretoria, South Africa; 3 Department of Family Medicine, University of Pretoria, South Africa; 4 School of Health Systems and Public Health, University of Pretoria, South Africa; 5 Department of Human Nutrition, University of Pretoria, South Africa; 6 School of Medicine, University of Pretoria, South Africa

## Introduction

Physical inactivity is the number one cause of most chronic diseases, and the largest health gains are achieved with increasing habitual physical activity. Exercise prescription is therefore an important part of patient counselling for healthcare professionals. Research suggests that doctors who engage in aerobic exercise are more likely to ask and advise their patients on physical activity. And medical students that engage in strenuous exercise perceive exercise counselling as highly relevant to future clinical practice. Therefore the physical activity habits of healthcare professionals is important, yet little has been published.

## Methods

This cross-sectional, observational study involved 281 consenting 1^st^ year MBChB and Bachelor of Clinical Medicine Practice (BCMP) students at University of Pretoria (100 male, 181 female, mean age 19.9±2.7 years). The total score from the Frequency Intensity Time Index of Kasari was used to classify current physical activity levels into Low, Moderate and High. Outcome variables included prevalence of physical activity levels and frequency of cardiorespiratory, strength and flexibility exercise. Chi square statistical test was performed to discern differences between male and female participants.

## Results

66% reported low levels of habitual physical activity (males 61%, females 68%; not significantly different (NS)), 16% report moderate levels (males 17%, females 16%; NS) and 19% report high levels of physical activity (males 22%, females 17%; NS). Approximately 70% of students report doing fewer than 3 days per week of cardiorespiratory exercise. 77% do <3 days per week of strength-related exercises and 86% do less than 3 days per week of flexibility exercise.

## Conclusions

The majority of 1^st^ year medical students in this study report engaging in low levels of physical activity. These preliminary data form part of research identifying lifestyle-related risk factors that should be addressed, thereby informing the development and implementation of the HEalthy Lifestyle Programme for HEALTHcare professionals (HELP-Health).

# Abstract: P05: The psycho-hormonal effects of aerobic fatigue on collegiate female soccer players

**Published:** 2019-01-01

**Authors:** Adele Broodryk, Cindy Pienaar, David Edwards, Martinique Sparks

**Affiliations:** 1 Physical Activity, Sport and Recreation Research Focus Area, Faculty of Health Sciences, North-West University, Potchefstroom, South-Africa; 2 Psychology Department, University of Zululand, Private Bag X1001, KwaDlangezwa, 3886, South Africa

## Introduction

Up to 95% of a soccer match entails aerobic actions that may cause fatigue. Little is known about the effects of fatigue on the hormonal and psychological states of female players.

## Methods

Cortisol values (saliva sample), state anxiety (Spielberger State-trait Anxiety Inventory) and mood scores [Incredibly Short Profile of Mood States (ISP), comprising six subscales and total mood disturbances (TMD)] of 43 female players (aged 22.0 ± 2.7 years) were taken immediately prior to, and 15 minutes after, an aerobic fatiguing test (AFT: Yo-Yo Intermittent Recovery test).

## Results

Cortisol increased (d = 0.7, p = 0.007) and ISP–confusion and ISP–vigour decreased (d = 0.5, p = 0.01 – 0.02). At pre-AFT, a slight positive relationship between cortisol and anxiety-absence (r = 0.3, P = 0.05) was seen. TMD consistently demonstrated a strong relationship with all ISP- and anxiety scores (r > 0.4, p < 0.01). Post-AFT results demonstrated a positive relationship between cortisol and BLa^−^ (r = 0.3, p = 0.04), ISP–anger with HR_max_ (r = 0.3, p = 0.03), YYIR-level and ISP-fatigue (r = 0.4, p = 0.04), and RPE with ISP–vigour (r = −0.4, p = 0.008) and ISP-fatigue (r = 0.3, p = 0.05).

## Conclusions

Fatigue caused by prolonged activity may be a greater physiological than psychological stressor, though both may affect soccer performance. We recommend training players to increase their aerobic capacity to ensure maximal quality match-time before fatigue and its subsequent adverse physiological and psychological effects set in.

# Abstract: P06: Effect of sports massage on lactic acid clearance during recovery from anaerobic exercise

**Published:** 2019-01-01

**Authors:** Robyn Burrows, Grant de Wet, Susan H Bassett

**Affiliations:** 1 Department of Sport, Recreation and Exercise Science, University of the Western Cape, Cape Town, South Africa

## Introduction

Research on the use of massage to enhance recovery has produced ambiguous findings in recent years. Athletes and physiotherapists typically believe that massage can aid recovery, but there is insufficient scientific evidence to support this thinking. During recovery after exercise, the body must restore the energy substrates it has used, remove metabolic waste products and repair the body’s tissues. Therefore, increased blood flow during recovery from high intensity exercise is important as it allows for the efficient transport of lactic acid from within the muscle via the blood to the heart. This allows the heart enzymes to convert lactic acid to pyruvate for further energy metabolism in the body. Therefore the aim of this study was to ascertain whether sports massage is an effective treatment modality for the clearance of lactic acid during recovery in university sports people.

## Methods

Twenty healthy university hockey and cricket players (aged 20–26 years) participated in the study. A 30s Wingate anaerobic test was conducted to ensure high levels of blood lactic acid was generated. The resistance of the cycle ergometer during the test was calculated as 0.75% of body weight. Participants warmed up on the Monark^®^ stationary bicycle at 60 rpm for 2 minutes (with intermittent bursts of rapid pedalling), followed by a 30 s rest, after which the Wingate test commenced. Blood lactic acid levels were measured immediately post-exercise, 2 minutes after active recovery was completed, and at 5 and 10 minutes into the massage and passive recovery interventions. Statistical analysis was conducted using descriptive statistics in the form of means and standard deviations in order to describe the data. The dependent variables were compared between the two groups using an independent t-test. The level of significance was set at p<0.05.

## Results

No significant differences were noted between the various lactic acid test phases, however, the massage group displayed a 0.9% decrease in blood lactic acid concentration from 2 minutes of active recovery to 5 minutes into the massage intervention and, similarly, the passive recovery group also showed a decrease of 0.8% in blood lactic acid concentrations during this period. From 5–10 minutes of recovery intervention, the massage group had a 1.1% decrease in blood lactic acid levels whereas the passive recovery group showed a 1% increase. Both groups showed a similar overall decline in blood lactic acid concentrations throughout the 10 minutes of the recovery interventions.

## Conclusion

Although no significant differences were observed in blood lactic acid concentrations after high intensity exercise following 10 minutes of leg massage or supine passive recovery with legs elevated, blood lactic acid reduced at a faster rate during passive recovery than massage. This is only an indication of muscle lactic acid and massage may be flushing the muscles of lactic acid more effectively, hence the higher blood lactic acid concentrations compared with active recovery. Therefore it can be concluded that either recovery modality could be considered to be effective for clearing blood lactic acid following high intensity anaerobic exercise.

# Abstract: P07: Comparative upper-quarter posture analysis of female adolescent freestyle swimmers and non-swimmers

**Published:** 2019-01-01

**Authors:** Carolin-Mari Botha, France Rossouw, Tanya C. dS. Camacho

**Affiliations:** 1 Department of Physiology, Division of Biokinetics & Sport Science, University of Pretoria, Pretoria, South Africa

## Introduction

The recurrent and demanding upper-quarter limb movement patterns imposed by freestyle swimming, may lead to adaptive muscle length and tension changes, predisposing adolescents swimmers to postural malalignment, pain and injury. The study aimed to record and quantify the static upper-quarter postural alignment of competitive female adolescent freestyle swimmers, and to compare their results to their non-swimming peers and to normative values.

## Methods

The evaluation group (EVAL) consisted of 35 competitive swimmers (age: 15±3 y; stature: 166.5±9.9 cm; body mass: 65.5±7.7 kg) and the control group (CON) of 36 peers (age: 15±3 y; stature: 164.2±6.7 cm; body mass: 62.1±9.1 kg). Sagittal posture was assessed by photographic posture analysis. Median ± interquartile range and inter-group differences were calculated utilising STATA 13 software.

## Results

Analysis showed significant differences (p = 0.00) between groups for all variables, with EVAL demonstrating restricted median scores for head-tilt angle (−8.7°), cervical angle (−13.3°), and protraction- and retraction angle (−24°) and a higher score for thoracic angle (+7.4°), when compared to CON. Furthermore, the median scores of EVAL deviated from accepted normative values for head-tilt angle (−4.6°), cervical angle (−16.5°), protraction- and retraction angle (−15°) and thoracic angle (+21.2°). Therefore, EVAL demonstrated all measurement deviations indicative of risk for forward head with rounded shoulder postural malalignment. However, two median scores demonstrated by CON also deviated from the norm indicating a risk for forward head (cervical angle −3.2°) and mid-thoracic kyphotic posture (thoracic angle +13.8°).

## Conclusions

Adolescents risk developing upper-quarter postural malalignment possibly from poor postural habits. In this group of participants, however, the risk for postural malalignment and its associated risk for future pain and injury, may have been exacerbated by years of freestyle swim training.

# Abstract: P08: Dermatological conditions in recreational athletes participating in a mass community-based running event

**Published:** 2019-01-01

**Authors:** I H Chauke, M Schwellnus, CC Grant, D A Ramagole, S Swanevelder, E Jordaan

**Affiliations:** 1 Sport, Exercise Medicine and Lifestyle Institute (SEMLI) & Section Sports Medicine, Faculty of Health Sciences, University of Pretoria, South Africa; 2 International Olympic Committee (IOC) Research Centre, Pretoria, South Africa; 3 Faculty of Health Sciences (Emeritus Professor), University of Cape Town, South Africa; 4 Biostatistics Unit, South African Medical Research Council, Cape Town, South Africa; 5 Statistics and Population Studies Department, University of the Western Cape, South Africa

## Introduction

Dermatological conditions experienced by runners are recognized to be of increasing significance because of high incidence rates noted at running events. It is therefore important to investigate the incidence rates and associated risk factors to implement preventative measures. The aim of this study was to determine the incidence of, and risk factors associated with dermatological complications in the Two Ocean’s half marathon (21.1km) and ultramarathon (56km) runners.

## Methods

Dermatological complications (defined as any runner with a skin complication during the race, requiring assessment by a clinician) were recorded over an 8-year period during the Two-Ocean marathon race event (2008 – 2015). A total of 153 206 runners (92 038 males and 61 168 females) participated.

## Results

Dermatological complications had an average incidence of 1.34/1000 race starters for all runners (95% CI: 1.10–1.63). The incidence for the 21.1km marathon were 1.2/1000 race starters (95% CI: 0.95–1.60) while the incidence for the 56km marathon averaged 1.51/1000 race starters (95% CI: 1.13–2.02). The highest incidence recorded was in the 56km race participants (2013) was 3.40/1000 race starters (95% CI: 2.38–4.86). The 21.1 km runners showed the highest incidence in 2014 (1.95/1000 race starters, 95% CI: 1.32–2.88). Multivariable risk factor analysis indicated that the specific year of observation was a significant risk factor in the Half marathon (p=0.014) and in the Ultra marathon (p<0.0001). This may indicate a link with environmental conditions. The multivariable analysis furthermore indicated that for the Half marathon, older age (51+years), (p=0.017) and for the Ultra marathon experience were significant risk factors (p=0.014).

## Conclusion

Prevention strategies to reduce the risk of dermatological complications should focus on older participants, inexperienced Ultra marathon runners and developing management strategies to negate the effect of adverse environmental conditions.

# Abstract: P09: Relationships between in- and post-match heart rate recovery and heart rate variability of African badminton players

**Published:** 2019-01-01

**Authors:** Ben Coetzee

**Affiliations:** 1 Physical Activity, Sport and Recreation Focus Area (PhASRec), Faculty of Health Sciences, North-West University, Potchefstroom Campus, Potchefstroom, South Africa

## Introduction

The purpose of the study was to determine relationships between in- and post-match heart rate variability (HRV) and heart rate recovery (HRR) of male, African, singles badminton players.

## Methods

Twenty-two male, African, singles badminton players (age: 23.3 ± 3.9 years) participated in the study. Fix Polar HR Transmitter Belts were used to record variations in beat-to-beat (R-R) intervals for rest periods between sets (in-match) and for 60 seconds directly after play had stopped (post-match) for 46 matches. Absolute and relative HRR were also calculated at 60 seconds post-set or -match.

## Results

Cluster analyses reduced HRV-related variables and HRR were entered into forward stepwise multiple regression analyses. The level of significance was set at p≤0.05. Natural logarithmic transformation of the square root of the mean squared differences between successive R-R intervals (Ln-RMSSD: Beta=0.57, p=0.08) and the very low band peak frequencies in hertz (Peak VLF Hz: Beta=−0.44, p=0.033) acted as significant predictors of both in- and post-match HRR. The high band peak frequencies in hertz (Peak HF Hz: Beta=−0.83, p=0.000) and the natural logarithmic transformation of high frequency relative power expressed as normalised units (Ln-HFnu: Beta=1.28, p=0.01) were also identified as significant predictors of in- and post-match HRR, respectively.

## Conclusions

Most of the parasympathetic nervous system-related HRV parameters are significantly associated with changes in HRR during and after badminton matches, although the direction of relationships are not always accurate. Therefore, the assumption that specific HRV-related variables always reflect activity of certain branches of the autonomic nervous system may be inaccurate.

# Abstract: P10: Tackle and ruck technical proficiency in rugby union and rugby league: a review

**Published:** 2019-01-01

**Authors:** Steve den Hollander, Chanda Ponce, Mike Lambert, Ben Jones, Sharief Hendricks

**Affiliations:** 1 Division of Exercise Science and Sports Medicine, Department of Human Biology, Faculty of Health Sciences, the University of Cape Town and the Sports Science Institute of South Africa, Cape Town, South Africa; 2 Health through Physical Activity, Lifestyle and Sport Research Centre (HPALS), Faculty of Health Sciences, the University of Cape Town, South Africa; 3 Western Province Rugby Academy, Cape Town, South Africa; 4 Carnegie Applied Rugby Research (CARR) Centre, Institute for Sport, Physical Activity and Leisure, Leeds Beckett University, Leeds, UK; 5 Yorkshire Carnegie Rugby Union Football Club, Leeds, UK; 6 Leeds Rhinos Rugby League Club, Leeds, UK; 7 England Performance Unit, The Rugby Football League, Leeds, UK; 8 School of Science and Technology, University of New England, NSW, Australia

## Introduction

Research on tackle and ruck technique proficiency has been shown to improve performance and reduce injury risks in rugby union (RU) and rugby league (RL). One of the main aims of this research is to provide coaches with information to inform practice. With that said, a complete review of the research on tackle and ruck contact technique to date has not been consolidated and synthesised in manner for rugby stakeholders to assimilate. Therefore, the purpose of this review was to consolidate and synthesise RU and RL studies on tackle and ruck contact technique for rugby stakeholders.

## Methods

Three electronic databases were searched for peer reviewed research studies which analysed any technical movement pattern of a player in the tackle, ball-carry into contact, or ruck. Forty-one papers were identified (19 in RL and 22 in RU).

## Results

Research in RL primarily focussed on identifying factors that impact tackling ability. Leaner, fitter players, with greater lower body strength, tended to have more proficient tackle technique. Experience and level of play were positively associated with tackling ability. Research in RU focussed on identifying tackle and ruck techniques associated with performance measures and injury outcomes. Eleven tackle techniques and five ball-carrier techniques were associated with both performance measures and injury outcomes. Only one study analysed ruck technique in RU, related to injury outcomes, and further research identifying safe and effective ruck technique is recommended.

## Conclusions

The findings of RU research support national injury prevention programmes that advocate that safe contact technique is also effective technique. Research in RL highlight the importance of developing tackle technique and physical qualities to allow players to allow players to progress to higher levels. Further research describing the relationship between tackle technique and performance measures and injury outcomes in RU and research identifying factors associated with tackle and ruck proficiency in RU is recommended.

# Abstract: P11: Head and neck injuries sustained during the super rugby tournament: a prospective study involving 69 781 training player-hours and 6 520 match player-hours

**Published:** 2019-01-01

**Authors:** C Du Plessis, CC Grant, M Schwellnus, A Jansen Van Rensburg, C Janse van Rensburg, E Jordaan

**Affiliations:** 1 Sport, Exercise Medicine and Lifestyle Institute (SEMLI) & Section Sports Medicine, Faculty of Health Sciences, University of Pretoria, South Africa; 2 International Olympic Committee (IOC) Research Centre, Pretoria, South Africa; 3 Faculty of Health Sciences (Emeritus Professor), University of Cape Town, South Africa; 4 Biostatistics Unit, South African Medical Research Council, Pretoria, South Africa; 5 Biostatistics Unit, South African Medical Research Council, Cape Town, South Africa; 6 Statistics and Population Studies Department, University of the Western Cape, South Africa

## Introduction

Professional rugby union is a high-intensity collision sport and is known to have a high risk of head and neck injury (HNI). The aim of this study was to determine the incidence rate of HNI during the 2013 – 2016 Super Rugby Tournaments, and identify the associated injury risk factors.

## Methods

All elite male South African rugby players (n=868) that participated during the 2013 – 2016 annual Super Rugby competition were included. Injury and exposure (days) data (training and match) was collected daily by each team physician, through a secure, web-based electronic platform.

## Results

Thirteen point three percent of players sustained HNI during the 4 year period of the tournament, with an overall incidence rate (IR/1000 player-hours; 95% CI) of 1.5 (1.2–1.8). Match HNI were significantly higher (16.4; 13.4–19.8) compared with training HNI (0.1; 0.0–0.2). The head/face region was more commonly injured (70.4%) than the neck/cervical spine area (29.6%) for all injuries, as well as during match play for both areas: 72% vs. 28% respectively. The majority of injuries were of minimal or mild severity (>57.4%). Moderate and severe injuries accounted for 35.7% and 7.0% of all injuries, respectively. In the head/neck region, match injury to the brain/CNS (53%) were nearly four times higher than injury to muscle/tendons (14%), joint/ligaments (9%), and skin or bone (11% each). Tackling was indicated as the main mechanism of injury in concussion injuries (52%), brain/CNS (50%) and head/neck (44%) injuries, followed by being tackled (25%, 24% and 19% respectively) and collision (14%, 15% and 13% respectively). Front line players had a higher incidence of overall HNI (front line=1.7; 1.3–2.1: backline= 0.3; 0.9–1.7) and match HNI (front line=18.7; 14.4–23.8: backline = 13.8; 9.9–18.7).

## Conclusion

The overall incidence of HNI in Super Rugby is 1.5/1000 hours play with a 10 times higher incidence during matches (16.4). Most HNI are to the brain/CNS and occur as a result of tackling or being tackled. Front line players are at a higher risk compared to backline players. Risk management structures need to be developed to reduce the risk of HNI in Super Rugby.

# Abstract: P12: Work-related barriers encountered by football medical team in Eswatini

**Published:** 2019-01-01

**Authors:** Adiele Dube, Chantell Gouws

**Affiliations:** 1 Department of Health Education, Southern Africa Nazarene University, Eswatini; 2 Department Human Movement Science, University of Zululand, South Africa

## Introduction

Football is among the most reported sport to incur a high rate of soft and hard tissue injuries in the world and eSwatini, in particular. The country has suffered deaths of junior and senior football players in the past two seasons consecutively. Research has shown that there is a high turnover of football team medical team in the country. Therefore, the current study aims to investigate work-related barriers encountered by the team medical team in the Kingdom of eSwatini.

## Methods

Twenty eight football team medics were purposively recruited to participate in this study. Data was collected through interviews and focus groups on the perceived hindrances to medical team management. Cronbach’s-alpha method was used and it yielded a reliability coefficient of 0.83.

## Results

It was identified that there was lack of support for team medics for premier, super and promotional leagues, unfair treatment to medics in terms of contractual system, inadequate salary/incentives, unfavorable working conditions, inadequate resources and pressure for players to return to play (from technical and management staff) as major barriers to effective job satisfaction for team medics. Lack of opportunities for promotion, lack of time, and family commitments were reported as the least barriers encountered by medics.

## Conclusion

It is recommended that the eSwatini Football Association, through the National Sports Medicine Executive Committee should consider these findings and develop remedial measures to improve the challenges team medics are facing. The association should also ensure that there is encouragement of more female team medics in the game of football.

# Abstract: P13: Blood pressure responses over 24-weeks of rehabilitation using robotic locomotor training and activity-based training in persons with spinal cord injury (SCI)

**Published:** 2019-01-01

**Authors:** Robert Evans, Claire Shackleton, Sacha West, Laurie Rauch, Wayne Derman, Yumna Albertus

**Affiliations:** 1 Division of Exercise Science and Sports Medicine, Department of Human Biology, Faculty of Health Science, University of Cape Town, Cape Town, Western Cape, South Africa; 2 Department of Sports Management, Cape Peninsula University of Technology, Cape Town, Western Cape, South Africa; 3 Institute of Sport and Exercise Medicine, Division of Orthopaedic Surgery, Faculty of Medicine and Health Sciences, University of Stellenbosch, Cape Town, South Africa; 4 IOC Research Centre, South Africa

## Research Objective

Evaluate the effect of robotic locomotor training (RLT) compared to activity-based training (ABT) on blood pressure responses in persons with SCI.

## Design

Randomized controlled trial.

## Setting

Therapy & Beyond Rehabilitation Centre - Walking with Brandon Foundation.

## Participants

Volunteer sample of 16 participants, aged 19 – 60 years, with chronic (>1 year) traumatic motor incomplete SCI (C1–C8; ASIA C-D) were randomized into RLT (n=8) and ABT (n=8) groups.

## Interventions

The RLT and ABT interventions involved 60-minute sessions, three times per week, over 24-weeks. RLT exclusively used the Ekso GT exoskeleton. ABT involved resistance, cardiovascular and flexibility training.

## Main Outcome Measures

Brachial and ankle blood pressure.

## Short-term results

Supine diastolic BP was well-regulated at 6-weeks in the RLT group whilst it significantly lowered in the ABT group (mean difference = 7.13mmHg; 95% CI, 0.42–13.83; p = 0.03). Doppler ankle BP showed a significant increase after exercise in the RLT group at 4-weeks (mean increase = 19.31mmHg; 95% CI, 4.35–34.28; p = 0.003).

## Long-term results

Post-exercise diastolic BP was reduced in the ABT group at 20-weeks (mean difference = 13.13mmHg; 95% CI, 0.01 – 26.24; p = 0.04). Post-exercise doppler ankle BP significantly increased in the ABT group at 16-weeks (mean increase = 19.56mmHg; 95% CI, 4.60–34.53; p = 0.002).

## Conclusions

RLT and ABT demonstrated positive effects on peripheral circulatory function, however these benefits occurred at different time points. RLT showed changes in BP within 6 weeks, whereas ABT showed changes in BP later during the intervention period (16–24 weeks). Long-term benefits of ABT may be attributed to the unique resistance training component of the intervention.

# Abstract: P14: The Epidemiology of injury and illness of Athletes at the Indian Ocean Island Games 2019

**Published:** 2019-01-01

**Authors:** Daniel Garnett, Adisha Bholah, Yannick D’Hotman, Krsnamoortee Sunassee, Jon Patricios, Benita Olivier, Saul Cobbing

**Affiliations:** 1 Physiotherapy Department, College of Health Sciences, University of KwaZulu Natal; 2 Comite d’Organisation de la 10^eme^ Edition des Jeux des Iles de l’Ocean Indien (COJI 2019), Mauritius; 3 The Wits Initiative for Sport and Health, Faculty of Health Sciences, University of the Witwatersrand; 4 Physiotherapy Department, Faculty of Health Sciences, University of the Witswatersrand

## Introduction

The 10^th^ Indian Ocean Island Games (IOIG) was held in Mauritius from the 19 to 28 July 2019, this multi-sport event occurs every four years with athletes from seven islands of the Indian Ocean. This study to establish the extent of injury and illness of participating athletes was the first in the Games’ 40-year history.

## Methods

This prospective cohort study recorded athlete injury and illness data by medical personnel from the visiting islands and the host country during 14 sporting codes at 16 venues. The injury and illness reporting form was adapted from versions used at recent Olympic Games.

## Results

In total, 1521 athletes (531 women and 990 men) reported 160 injuries and 85 illnesses. The age of patients ranged from 10 to 59 years (mean: 25.21 years). An injury incidence of 12% and illness incidence of 6% were recorded. The highest number of injuries was documented in football (24%) and basketball (14%). Men suffered the majority of injuries (79% vs 21%), with most injuries occurring during matches/heats compared with training (57% vs 43%). The lower limb (63%) was most frequently injured with the most common sites being the thigh (20%), knee (18%) and calf (11%). Joint sprains were the most common type of injury (27%), followed by muscle strains (20%). The highest number of injuries was sustained from contact with other athletes (28%). The highest incidence of illness occurred in athletes participating in athletics (21%) and volleyball (15%) and men reported more illness than women (57% vs 43%). The majority of all illnesses affected the respiratory system (67%) and infection was the most common cause of illness (84%).

## Conclusion

At least 12% of all athletes sustained an injury and 6% sustained an illness during the Games. Analyses of illness risk factors and injury mechanisms is essential to direct future athlete welfare policies.

# Abstract: P15: A description of cardiovascular disease risk factors in a sample of factory workers in Johannesburg

**Published:** 2019-01-01

**Authors:** Dr Philippe Gradidge

**Affiliations:** Centre for Exercise Science and Sports Medicine, University of the Witwatersrand

The aim of this cross-sectional study was to describe the cardiovascular disease risk factors of a sample of factory workers in Johannesburg, South Africa.

A sample of 145 employees out of a potential 220 volunteered to participate. Point-of-care glucose, total cholesterol, blood pressure, and anthropometry were collected. Sociodemographic information, self-reported sleep, physical activity, and mental health were determined using questionnaires. Descriptive analyses were used to compare male and females participants. Females were younger in age, had higher body mass indices, but showed lower cardiovascular risk than males (p0.005 for these measures). Hypertension and depression were noted in ~30% of males and females, with high levels of stress in the study population. A high proportion of both males and females were current smokers and consumed alcohol regularly. The consumption of fruits and vegetables were relatively high in the sample, and most perceived themselves as physically active. In conclusion, this descriptive study has shown that the presence of cardiovascular diseases risk factors are high in active factory workers, despite adherence to protective behaviours.

# Abstract: P16:The Ukumela impilo study to interrupt sedentary behavior and improve cardiovascular health

**Published:** 2019-01-01

**Authors:** Dr Philippe Gradidge

**Affiliations:** Centre for Exercise Science and Sports Medicine, University of the Witwatersrand

The Ukumela impilo study is a randomised controlled trial based in at a company in Johannesburg. Phase one of the study aims to understand the sedentary behaviour and correlates of cardiometabolic diseases in a sample of office based workers. The sample will then be randomly assigned into one of two potential intervention groups and a control group, including a standing desk group, a group receiving health messages, and the control group. In phase two, follow-up testing will take place post-12-week intervention to determine the changes in sedentary behaviour and cardiometabolic diseases. Focus groups will be conducted in phase 3 of the study to determine the experiences of the sample engaged in the two intervention groups. The purpose of this paper is to describe the development of a conceptual model to test the efficacy of the two interventions in the office-based workers. In conclusion, the researchers, in consultation with stakeholders, will formulate a sustainable model for addressing extended sitting in office-based workers.

# Abstract: P17: The effects of foam rolling and stretching as recovery tools on power output, mood state, muscle stiffness, and muscle pain

**Published:** 2019-01-01

**Authors:** M Claassens, HV Hammill

**Affiliations:** 1 Department of Biokinetics, University of Zululand, South Africa; 2 School for Human Movement Sciences, North-West University, Potchefstroom Campus, South Africa (PhASRec)

## Introduction

Recreational sportsmen strive to improve performance while increasing their training load. Muscle pain and -stiffness can occur after an increase in exercise intensity and volume, after eccentric muscle contraction. When recreational sportsmen persevere in training whilst experiencing muscle pain, poor coordination and mood state can be experienced; which can lead to a reduction in muscle strength. Methods that have been used to decrease muscle pain, such as massage and vibration therapy are expensive and impractical. This study aims to discover an inexpensive recovery tool that decreases muscle pain and -stiffness whilst simultaneously improving muscle strength and mood state. Foam rolling (FR) and static stretching (SS) are considered possible suitable recovery tools.

## Methods

Sixty-seven (N=67) healthy, recreational sportsmen were recruited from sport clubs in Kwa-Zulu Natal to voluntarily participate. Participants were divided into: The FR group (n=23), the SS group (n=25) and a control group (n=19). Power output [maximum voluntary concentric (MVC)] and mood state [profile of mood status (POMS)] were measured on all participants at baseline, after which muscle pain and -stiffness were induced (through eccentric hamstring exercise session). Power output and mood state were then measured 48 hours post eccentric exercise session. Following these measurements the FR and the SS groups commenced with a three-day intervention, performing either FR or SS on the hamstring. The control group performed no intervention. Muscle pain and -stiffness were measured on all participants at baseline, three times a day for two days post intervention. Mood state was measured 24 hours and 48 hours after the eccentric exercise. An Analysis of Variance (ANOVA) was conducted with a significance level of ≤0.05.

## Results

Both FR and SS significantly reduce eccentrically induced hamstring muscle pain (p<0.05). No statistical difference in muscle stiffness between groups was recorded. The FR group’s muscle stiffness is continuously lower than that of the SS and control group. In all groups, vigour, a component of mood state, decreased over the course of the intervention. While this means that a decrease in performance can be expected, no significant decrease was found between groups. This can be due to numerous external stress factors experienced by each participant during the day. The FR group reveals a strength output loss (p<0.31), whereas the control group (p<0.000) and the SS group (p<0.002) reveals a more significant strength output loss. Evidence suggests that using FR compared to SS as a recovery tool will be of greater benefit as concerns the improvement of strength output. Recreational sportsmen can thus strive to achieve optimal performance whilst continuously increasing training.

## Conclusions

FR and SS are safe, practical, and inexpensive tools that can assist in decreasing muscle pain after a strenuous bout of eccentric exercise. Although SS is historically a widely used recovery tool, it may not be the best tool for the improvement of performance. Only FR was shown to prevent strength loss and improve muscle stiffness as compared to SS and control group.

# Abstract: P18: “I am active and healthy, so I don’t need to make lifestyle changes!” A short report of clinical markers of “risk” for NCD’s versus health and physical activity perceptions in a low-resourced setting.”

**Published:** 2019-01-01

**Authors:** Brittany Fell, Ashleigh Robinson, Susan Hanekom, Wayne Derman, Martin Heine

**Affiliations:** 1 Institute of Sport and Exercise Medicine, Faculty of Medicine and Health Sciences, Stellenbosch University; 2 Division of Physiotherapy, Faculty of Medicine and Health Sciences, Stellenbosch University; 3 IOC Research Centre, Cape Town, South Africa

## Introduction

It is important to understand how to facilitate behaviour change in the context of healthy lifestyle, where gratification is often delayed and not directly noticeable, to prevent and manage NCD risk factors. The objective of this cross-sectional and pragmatic study was to determine clinical markers of NCD risk in a low-resourced and socio-economic challenging neighbourhood, and to determine the association between key commonly measured health parameters with self-reported levels of physical activity and perceived health.

## Methods

Bishop Lavis (Cape Town, South Africa) is a low-resourced and socio-economic challenging neighbourhood. All participants of the annual Bishop Lavis health festival (Friday 19th of October 2018) were requested to complete a fully anonymous “health passport”, which included gender, age, smoking, alcohol, drug-use, work status, medical insurance, and self-reported presence of non-communicable disease (i.e. Cancer, Cardiovascular Disease, Respiratory disease or Diabetes). Perceived levels of current physical activity and perception of health were recorded using a simple Likert scale ranging from 1 (unhealthy; inactive) to 5 (very healthy, very active).

## Results

Out of the 104 participants, 63% presented with blood pressure recordings indicative of hypertension. A majority of the participants (58.1%) could be categorised as obese (class I–III). Non-fasting blood glucose concentrations could be considered normal in most participants (81.6%). Participants reported on average a 4.1 (±1.0) in terms of engaging in habitual physical activity, and 4.0 (±1.1) in terms of health on a 5-point Likert scale. Only the bivariate association between systolic blood pressure and reported physical activity was found significant (p<0.05).

## Conclusions

This pragmatic study exemplifies that these participants, living in a challenging socio-economic context, portrait a clinical risk profile in line with that for the development and presence of NCD. However, these risks did not reflect in point-estimates of perceived physical activity and health.

# Abstract: P19: Performance and anthropometrical differences between sexes at the onset of puberty

**Published:** 2019-01-01

**Authors:** Charne Scott, Kirsty Elliott, James Clark, Cassidy Grobler, Mariska Fisher, Charl Janse van Rensburg

**Affiliations:** 1 Sport, Exercise Medicine and Lifestyle Institute (SEMLI) and Division of Biokinetics and Sports; 2 Science, Department of Physiology, Faculty of Health Sciences, University of Pretoria; 3 Department of Sport and Leisure Studies, University of Pretoria; 4 Biostatistics Unit, South African Medical Research Council

## Introduction

Understanding anthropometric and physical performance capability between youths of similar maturational status (pre-pubertal) but in different sexes, is needed to guide professionals in the prescription of exercise and sport. The aim of this study was to compare anthropometric and physical performance parameters in pre-pubertal (pre-peak height velocity; pre-PHV) girls and boys.

## Methods

62 active youths (age = 11.7 ± 1.0 yrs.; females = 37, males = 25), were classified as pre-PHV, according to maturity status (Mirwald equation). Anthropometric measures (height, weight, body fat percentage, arm span, leg length, seated height) and physical performance tests (grip strength, vertical jump, prone bridge, modified sit and reach) were compared between girls and boys with the same maturity status.

## Results

Chronological age and anthropometric measures (height, weight, absolute arm span, absolute and relative seated height and absolute and relative leg length) were significantly higher in boys compared to girls (p=0.05), but body fat percentage was not different between girls and boys. Within the performance tests 1) boys scored significantly higher in absolute grip strength (R, p=0.00; L, p=0.00) absolute vertical jump height (p=0.008), and relative grip strength (kg/kg; mean ± SD)(left) (boys = 0.52 ± 0.12; girls = 0.45 ± 0.09; p=0.018), 2) girls scored significantly better than boys in absolute modified sit-and-reach test (p=0.05), and relative to stature (boys = 0.19 ± 0.03; girls = 0.24 ± 0.07; (cm/cm) p=0.01), and 3) there was no significant between-sex difference in relative vertical jump height (p=0.07) and absolute prone bridge time to exhaustion (p=0.29).

## Conclusions

In absolute performance measures there were significant differences between prepubertal boys and girls, and therefore, prescription of exercise and sport should be tailored accordingly.

# Abstract: P20: The kinetics and kinematics of the rotary backlift

**Published:** 2019-01-01

**Authors:** Neil Isaacs

**Affiliations:** Private General Practice, Ballito, South Africa

## Background

Video analysis has revolutionized coaching with slow motion replays now becoming available to anyone with cell phone capability of 200 frames per second. The era of pre and post video analysis has closely coincided with increased interest in the rotary batting technique with highlighted findings of a more lateral backlift.

## Aims

A review of the backlift by slow motion video analysis of Don Bradman and modern top international ranked players to obtain aspects of the rotary batting technique with regard to functional anatomy related to rotations around the long axis of the limbs, trunk and bat with comparison to traditional coaching.

## Methods

This is a descriptive observational study. Slow-motion videos of footage of Don Bradman and replays of top international batsman were reviewed by taping the slow-motion videos of the backlift in turn with the slow-motion function of an Apple iPhone7.

## Results

Backlift loop divided into stages:

### Stage 1

The backlift of Don Bradman involved flexion of the elbows from pointing the face of the bat to midwicket in the stance, to lifting the bat horizontally pointing to gully by extending the shoulders and flexing the elbows till the bat was in the same horizontal plane of the elbows.

### Stage 2

The front shoulder internally rotates, using the back hand as a fulcrum, the bat lifts into a vertical position as the back wrist cocks (radial deviation), the back shoulder extends with the back forearm still directed towards cover. The backlift may end in Stage 2 or continue to any point at the end of stage 3.

### Stage 3

Abduction of the back shoulder elevates the hands and forearm to the level of the back shoulder with the bat still vertical, maintaining the forearm and bat face directed to point, the back forearm supinates, keeping the bat vertical.

From the stance to the top of the backlift, the edges of the bat were directed in the plane of the pitch. External rotation of the front hip may initiate rotation and closing of the hips and shoulders.

## Discussion and Conclusions

During stage 1, the centre of mass (COM) shifts towards gully. Preparation for the stride starts during Stage1.Moment of inertia (MOI) of the arms and bat is reduced by extension of the back shoulder and flexion of the elbow. At the end of all stages, the hands are further to the off side compared to traditional coaching, allowing flexibility. Forward strides keep the bat close to the COM, optionally moving the hands forwards upwards and backwards in a semicircle, aiding propulsion in the direction of the stride. Similarly, backward strides rotate the hands backwards and upwards. Sideways movement to the off side is aided by flexing the back shoulder and flexion or extension enables flexibility of the vertical pendulum line of the downswing. Traditional coaching and studies reveal an increased MOI, the elbow above the back hand and the back shoulder internally rotated to form a figure 6 with the arms at the end of the backlift.

# Abstract: P21: The kinetics and kinematics of the rotary downswing in batting

**Published:** 2019-01-01

**Authors:** Neil Isaacs

**Affiliations:** Private General Practice, Ballito, South Africa

## Background

Research on cricket batting is limited with mainly descriptive studies. Findings of a more lateral backlift in the best players of the last century against traditional coaching of a straighter backlift towards the stumps raises the question as to the reason why.

## Aims

A review of the downswing by slow motion video analysis of Don Bradman and modern top international ranked players to obtain aspects of the rotary batting technique around the long axis of the limbs and trunk and related functional anatomy to improve the velocity of the bat (performance) compared to traditional coaching.

## Methods

This is a descriptive observational study. Slow motion videos of footage of Don Bradman and replays of top international batsman were reviewed by taping the slow-motion videos of the downswing in turn with the slow-motion function of an Apple iPhone7.

## Results

Downswing stages:

### Stage 1

The back hip horizontally abducts while flexed up to 90 degrees, after the pelvis closed and the back hip adducted with a varus or neutral back knee in the rotary backlift, opening the pelvis. Shoulders close, back shoulder externally rotates and adducts the elbow to the waist. Back forearm supinates and wrist extends obtaining the bat in the direction of the pendulum swing until the bat is horizontal.

### Stage 2

Back hip externally rotates while the shoulders open towards direction of shot, after X-factor stretch, passively externally rotating the back shoulder. The back elbow remains close to the waist, forearm supinated and wrist extended.

Double pendulum formed by front arm and bat is released as late as possible.

Bat face is directed to point in stage 1 and 2 in vertical pendulum shots.

### Stage 3

Active internal rotation of back upper arm occurs immediately before impact, after horizontal adduction accelerates the back upper arm, still externally rotated, into the scapula plane. Pronation of the back forearm, flexion and ulnar deviation of wrist occurs through impact. Front foot and back foot are in the direction of the shot in front and back foot vertical pendulum shots respectively.

## Discussion and Conclusions

Stretch reflexes of the back hip, trunk and back shoulder add power to the shot. At impact the hips and shoulders are open compared to shoulders rotating vertically in traditional coaching. Back upper arm moves from external rotation to internal rotation compared to being internally rotated from top of backswing to impact in traditional coaching. “O” grip noted increases moment of inertia around back upper arm and with opening of the shoulders, concern of deviation off the line of the downswing to the off side in vertical pendulum shots and above the horizontal pendulum in cross bat shots is allayed. Lateral flexion occurs at the lumbar spine to increase the thoracohumeral angle closer to 90 degrees increasing the bat velocity. Impact is away from the front foot in front foot drives and back foot in backfoot drives.

# Abstract: P22: Exercise may decrease syncope secondary to postural change in females with rheumatoid arthritis: Pilot study

**Published:** 2019-01-01

**Authors:** Dina C (Christa) Janse van Rensburg, JA Ker, CC Grant, L Fletcher

**Affiliations:** 1 Section Sports Medicine, Faculty of Health Sciences, University of Pretoria, Pretoria, South Africa; 2 Internal Medicine, Faculty of Health Sciences, University of Pretoria, South Africa; 3 Department of Statistics, Faculty of Natural and Agricultural Sciences, University of Pretoria, Pretoria, South Africa

## Introduction

The autonomic nervous system (ANS) regulates heart rate via sympathetic and parasympathetic influences. Rheumatoid arthritis (RA) patients suffer from autonomic dysfunction, which may consequently lead to syncope with possible falls after posture change i.e. rising from supine to standing position. Previous research has shown general ANS improvement after exercise, but not in specific relation with posture change. The aim of this study was to determine the effect of exercise on posture change (supine to standing) in females with RA as measured by short-term heart rate variability (ANS function).

## Methods

Patients with confirmed RA were randomly selected to a control- (RAC) or exercise group (RAE). RAE group (n=19) trained 2–3 times/ week under supervision. RAC group (n=18) continued their current sedentary lifestyle. The medium intensity exercise programme lasted 12 weeks. No medication change was allowed during this time. ANS function and balance were determined by quantification of the inter-beat interval variability, with the Polar 810i heart rate monitor system. Frequency domain analyses were used for quantification: LF (ms_2) – mainly sympathetic influence; HF (ms_2) – parasympathetic influence; and LF/HF – autonomic balance.

## Results

The two groups’ baseline demographic data (age, sex, disease activity, disease duration) matched. Comparing pre- to post posture change intervention (i.e. standing value minus supine value), all frequency domain parameters changed as anticipated (i.e. vagal withdrawal and increased sympathetic influence) for the RAE group. The RAC group’s measurements deteriorated: LF (ms_2): RAE −1.03 to 22.03 (stronger sympathetic influence); RAC 43.45 to −31.21 (weaker sympathetic influence); HF (ms_2): RAE −24.03 to −33.34 (better vagal withdrawal); RAC −191.7 to −114.1 (less vagal withdrawal); LF/HF: RAE 10.57 to 15.04; RAC 2.9 to 7.6.

## Conclusion

Preliminary results indicate that exercise may indeed improve autonomic function in RA patients, in such a way that posture change will not be an added burden for falls in an already otherwise compromised population.

# Abstract: P23: Exploring the efficacy of low-level laser therapy and exercise for knee osteoarthritis

**Published:** 2019-01-01

**Authors:** A Kholvadia, D Constantinou, P J-L Gradidge

**Keywords:** photobiomodulation, physical therapy, degenerative joint disease

## Background

Knee Osteoarthritis (KOA) is a prevalent, chronic disorder with excessive functional, social and economic burdens. The goal of treatment is to alleviate the symptoms and slow the progression. Documenting the effects of exercise and LLLT as co-modalities in the management of KOA allows practitioners to implement this management tool as part of KOA rehabilitation, resulting in the earlier discharge from a supervised rehabilitation setting.

## Objective

The purpose of this study was to determine the effect of low-level laser therapy (LLLT) in the treatment of knee osteoarthritis (KOA). A randomised controlled trial (RCT) was conducted on 111 participants (aged between 40–75 years) diagnosed with KOA. Participants were randomised into an exercise (n=39), LLLT (n=40), or a combined exercise-LLLT (n=32) group.

## Methods

The Western Ontario and McMaster Universities Osteoarthritis Index (WOMAC) scale was used to assess pain and functionality. Knee range of motion was assessed using a goniometer, and the one-minute timed sit–to-stand test measured physical functionality at four time points: (T1) baseline, (T2) post 12-session intervention, (T3) one-month post intervention and (T4) three-month’s post intervention. Knee circumference was measured using a measuring tape.

## Results

WOMAC pain and functionality scale and knee circumference scores decreased in all three groups (P<0.05), but the combined exercise-LLLT group demonstrated better outcomes than the LLLT or exercise alone groups respectively. The combined exercise-LLLT group showed better acute and long-term benefits with participants experiencing a 3.5 centimetre decrease in knee circumference, 24 point improvement in the WOMAC pain and functionality scale, and a four repetition increase in physical functionality.

## Conclusion

The findings suggest that LLLT is a viable tool for managing KOA when used in conjunction with physical exercise.

# Abstract: P24: Altered thoracic spine kinematics as a potential cause of low back pain in rotational-sport athletes: a case study

**Published:** 2019-01-01

**Authors:** Dimitrije Kovac, Ranel Venter

**Affiliations:** 1 Fitness Medico, Belgrade, Serbia; 2 Department of Sport Science, Stellenbosch University, South Africa

## Introduction

It has been proposed that an excessive, compensatory, motions of the lumbar vertebrae, often caused by limited mobility of the thoracic vertebral segments, can induce low back pain (LBP). Such altered kinematics in rotational sports (i.e. tennis, golf and baseball), may explain the high prevalence of low back pain in this athletic population.

## Methods

We report the case of an 18-years old male tennis player (height 180cm, weight 69 kg, right-handed) with LBP affecting sport performance. The patient reported a six-month lasting LBP which compelled him to stop training and playing matches several times. He was treated with different modalities of electrotherapy, ultrasound, manual mobilization and manipulation to the lumbar spine, massage and stretching with only temporary relief of symptoms. He was not able to compete on his previous level without symptoms. Magnetic Resonance Imaging (MRI) of the lumbar spines showed no pathological signs. A functional examination with the seated rotation test (bar in front of body) was done. The patient was seated, with hips and knees flexed to 90° and a foam roller placed between the knees to minimize motion of the lower extremities and lumbar spine during thoracic rotation. A 100 cm-Iong, 2-cm-diameter wooden stick, placed on chest, was used to standardize the position of the upper extremity. The patient was then asked to rotate his trunk to each side. A 0–3 grading scale from the nine test screening battery firstly described by Frohm et al. (2012) was used. If the stick did not reach patient’s knee on both sides, a score of 1 was given bilaterally. If the bar was in line with the knee, the score for that side would be 2. If the bar was in line with the ball the score of 3 would be given. High intertester (0.87) and intratester (0.87) reliability was previously reported for the test. Two exercises („Rib-grab rotation”, „T-spine rotation with reach”) that focused specifically on thoracic spinal rotation were added to his regular strength and conditioning programme. The exercises were done bilaterally with 3 sets of 6 repetitions. Stabilization of the lumbar spine was emphasized during thoracic rotations. During the intervention period the player was allowed participate in some tennis sessions without provoking symptoms.

## Results and conclusion

Following the six-week intervention period, a significant improvement in thoracic spine rotation was noted with a score of 3 for both sides on the seated rotation test. The patient also reported significant reduction in pain and he was able to return to high level of training and competition. Six months later, the patient maintained the same level of thoracic spine rotation and reported a complete reduction in symptoms.

# Abstract: P25: A five year clinical audit of concussive injuries in collegiate male rugby players- a Stellenbosch University experience

**Published:** 2019-01-01

**Authors:** L. Kunorozva, J. C. Brown, P. L. Viviers, E. W. Derman

**Affiliations:** 1 Institute of Sport and Exercise Medicine, Division of Orthopaedics, Faculty of Health Sciences, Stellenbosch University, Stellenbosch, South Africa; 2 Campus Health Service, Stellenbosch University, Stellenbosch, South Africa; 3 IOC Research Centre, South Africa

## Background

Sports-related concussion (SRC) is a common injury occurring mostly in contact sports. More specifically, SRC is a salient health concern for male collegiate rugby athletes because at least one concussion is reported in every two games. Thus, a regular clinical audit of concussive head injuries is warranted in order to provide objective data pertaining to the injury so that pattern recognition can be studied.

## Aims

The purpose of this study was to clinically analyze and report on the concussion rate and symptom patterns from concussive head injuries in collegiate male rugby players for the period 2013–2018.

## Methods

A retrospective analysis of head injuries in a cohort of Stellenbosch University collegiate male rugby players (n=367), age (mean±SD; 24.6±4.3), height (mean±SD; 1.83±0.07), weight (mean±SD; 90.7±14.4) and BMI (mean±SD; 26.5±5.7). Each of the players with a suspected concussion returned for a clinical assessment performed using the Sports Concussion Assessment Tool (SCAT)-3 (n=252) and/or SCAT-5 (n=45). The SCAT questionnaire evaluates both cognitive and physical symptoms based on a scale of 0–6, where 0 indicates the absence of symptoms, while 6 indicates the highest severity. The symptoms and symptom scores are added to give a symptom (of 22) and severity (of 132) scores, respectively.

## Results

The concussion incidence rate was 7.3% of total registered athletes per year representing 87 concussions per year. Of the reported symptoms, headaches (57.1%) and pressure in head (55.8%) were the most frequently reported with sadness (12.7%) the least reported. The third SCAT mean symptom (3.2/22) and severity (5.7/132) scores were comparable to the sixth SCAT mean symptom (2.3/22) and severity (2.5/132) scores post injury. Thirty percent of the 434 head injuries had no SCAT information.

## Conclusion

The results of this study indicate i) On average 87 concussions per year were treated by the medical staff; II) headache and pressure in head are the most common presenting symptoms associated with concussion in collegiate rugby players and iii) these findings provide valuable information for planning purposes as well as players education.

# Abstract: P26: Creating an evidence-based decision-making algorithm for the individualised and holistic management patellofemoral pain

**Published:** 2019-01-01

**Authors:** Dr Dominique Leibbrandt, Prof Quinette Louw

**Affiliations:** Division of Physiotherapy, Department of Health and Rehabilitation Sciences, Stellenbosch University

## Introduction

Patellofemoral pain (PFP) is a multifactorial condition with unknown aetiology. Recent studies have suggested that there are subgroups of individuals with PFP who present differently and respond differently to treatment. Therefore, the purpose of this presentation is to use the best available evidence on risk factors for patellofemoral pain to create a holistic decision-making clinical algorithm that provides recommendations for the most important components to consider for the individualised management of the condition.

## Methods

A search was conducted in PubMed, Scopus and Science Direct for all systematic reviews investigating risk factors or contributing factors for patellofemoral pain. Using these reviews, the different categories of risk factors were identified, and the factors supported by the evidence were extracted. This was then used to create an algorithm for clinicians to use to screen for risk factors that they should consider first in treatment. The algorithm also includes treatment options that can be used to address the specific factors that individuals with PFP may present with. This algorithm can be used as a guideline to assist clinicians with treatment planning and individualised management.

## Results and conclusion

Six systematic reviews were included. The risk factors associated with PFP include biomechanical risk factors during walking, running and squatting; muscle strength; body mass index and psychological risk factors. Sub-groups of subjects with patellofemoral pain include adults, females, military recruits, recreational runners and adolescents. An individualised approach to assessment and treatment is needed for the management of PFP. Treatment recommendations should consider different subgroups of individuals with PFP as the risk factors are different amongst sub-groups. Future research should incorporate expert opinion of clinicians for the management of PFP as this will improve our understanding of what works and unable us to identify factors that could be important but aren’t currently identified due to a lack of evidence

# Abstract: P27: Experiences of young South African gymnasts, parents and coaches about the health benefits of sport participation

**Published:** 2019-01-01

**Authors:** WC Lucas, S Titus, MEM Young

**Affiliations:** 1 Department of Sport, Recreation and Exercise Science, University of the Western Cape, Cape Town, South Africa; 2 Alcohol, Tobacco and Other Drugs Research Unit, South African Medical Research Council, Cape Town, South Africa; 3 Interdisciplinary Teaching and Learning Unit, School of Public Health, University of the Western Cape, Cape Town, South Africa

**Keywords:** Gymnastics, parents, coaches, sport participation, physical, cognitive and socioemotional benefits

## Introduction

Gymnastics, as a physical activity, presents young people with opportunities to learn physical skills, improve eye-hand coordination, develop gross and fine motor skills, and socially develop through engagement with other young people. An understanding of aerobic and anaerobic physical activities is important to enhance young people’s physical, emotional, cognitive and social development.

## Methods

This study investigated the perceptions of a purposively sampled group of young people in the Western Cape of South Africa about the health benefits of gymnastics participation, and reported findings from the perspectives of registered competitive gymnasts, gymnastics coaches, and parents of gymnasts (total n=34), with specific reference to the domains of physical, cognitive and socio-emotional development. A qualitative approach based on the Process-Person-Context-Time (PPCT) concept, adapted from the bioecological model, was used as the theoretical framework to underpin, analyze and interpret findings of the study. Four key informant interviews were conducted with four coaches, and five focus group discussions were held with gymnasts, parents of gymnasts and gymnastics coaches.

## Results

Thematic analysis of the participants’ responses indicated the following themes as health benefits and opportunities for children’s development through gymnastics participation: socio-emotional development with respect to positive attitude shifts, respect of peers and coaches, and physical development concerning musculoskeletal growth.

## Conclusion

Development of strength and flexibility were also found to be positive contributors to child development. Additionally, improvements in cognition which could positively influence children’s academic achievement and the learning of new movement skills, were also attributed to gymnastics participation. The implications of the findings for children’s holistic development was discussed.

# Abstract: P28: The epidemiology of iliotibial band friction syndrome (ITBFS), and risk factors associated with ITBFS, in 21.1km and 56km distance Runners

**Published:** 2019-01-01

**Authors:** Fhulufhelo Madzhiga, A Jansen van Rensburg, Martin P. Schwellnus, Dina C (Christa) Janse Van Rensburg, Sonja Swanevelder, Esme Jordaan

**Affiliations:** 1 Sport Exercise Medicine and Lifestyle Institute (SEMLI) and Section Sports Medicine, Faculty of Health Sciences, University of Pretoria, Pretoria, South Africa; 2 Emeritus Professor of Sport and Exercise Medicine, Faculty of Health Sciences, University of Cape Town, South Africa; 3 International Olympic Committee (IOC) Research Centre, South Africa; 4 Biostatistics Unit, Medical Research Council, Parow, South Africa; 5 Statistics and Population Studies Department, University of the Western Cape

## Introduction

Iliotibial band friction syndrome (ITBFS) is the most common overuse injury affecting the lateral aspect of the knee in distance runners. Novel factors such as medical conditions and medication use are related to other overuse injuries in runners, however, there is limited data on their link to ITBFS. The aim of this study was to identify the risk factors associated with ITBFS in distance runners.

## Methods

Participants (n=12 659) in the Two Oceans races (21.1km, and 56km) completed a pre-race medical history screening tool including: training, cardiovascular disease (CVD), risk factors for, and symptoms of CVD, history of diseases affecting major organ systems, cancer, allergies, medication use, and running injury. Runners were grouped as having a past history of ITBFS (hITBFS group n=509) and a control group (n=12 150).

## Results

Independent risk factors associated with a higher risk of a history of iliotibial band friction syndrome (hITBFS) compared to runners with no history of these factors were as follows: any risk factors for CVD (PR=1.5; p=0.0002); any respiratory disease (PR=1.5; p=0.0004); any endocrine disease (PR=1.7;p=0.012); any GIT disease (PR=2.7; p<0.0001); any nervous system psychiatric disease (PR=1.8; p=0.005); a history of any allergies (PR=1.7; P<0.0001); the use of any analgesic anti-inflammatory medication (AAIM) during a race or in the week before a race (PR=5.8; p<0.0001) and intermediate category of runners (PR=1.4; 95%CI: 1.1–1.8).

## Conclusion

Novel independent risk factors associated with ITBFS in long distance runners were participation in running for <3 years, being an average runner, having any risk factor for CVD, having a history of disease (i.e. respiratory, endocrine, GIT, central nervous system CNS), allergies and the use of AAIM medication a week prior to the race. Future longitudinal research is needed to assess the cause effect of these risk factors during distance running and for developing preventative strategies.

# Abstract: P29: The development of the C.R.E.S.T anti-doping protocol

**Published:** 2019-01-01

**Authors:** Thanujj Kisten, Rowena Naidoo

**Affiliations:** Discipline of Biokinetics, Exercise and Leisure Sciences, University of Kwa-Zulu Natal, Westville campus, South Africa

## Introduction

The purpose of this study is to develop an anti-doping protocol to promote positive behaviour change in U-17 soccer players. The protocol is guided by the Chaos Theory. This theory provides a valid framework when studying behaviour change. The theory purports that behaviour change occurs in quantum leaps and is not in a linear, deterministic process. Hence, the development of the protocol required different components to allow for different starting points.

## Methods

This study employed a qualitative research approach with focus group discussions. Experts in the field of sport psychology, physics, value-based education, anti-doping education and sports science were identified to contribute in the development of the protocol. Knowledge from the various experts were integrated, together with literature in the field of anti-doping protocols/interventions, were used to develop the anti-doping protocol.

## Results

The following five components for an anti-doping protocol were developed; Coping techniques (C), Relaxation techniques (R), Education (E), Skills (S), Training (T) - C.R.E.S.T. Each component comprised the following activities; Coping (various activities were recommended, specifically activities that involved humour), Relaxation (breathing exercises, yoga and imagery), Education (The South African Institute for Drug-Free Sports (SAIDS) educational material, Skills (sport specific skills), and Training (physical fitness activities). Each C.R.E.S.T session will be 60 minutes with approximately 12 minutes per component. Each of these components are independent, but inter-dependent on one another.

## Conclusions

In order to determine whether the protocol has an impact on behaviour change, it will be tested in future intervention studies. To test the validity of the Chaos Theory, the C.R.E.S.T protocol will be implemented, beginning at the various starting points, i.e. with a different component. This anti-doping protocol will be the first of its kind. Additionally, the use of the Chaos Theory is an innovative, yet complex method to promote behaviour, but since behaviour change is a complex process, this theory deems appropriate.

# Abstract: P30: Research on the supply of public sports service in Chinese sports parks

**Published:** 2019-01-01

**Authors:** Wei Qian

**Affiliations:** College of Physical education, Soochow University, 2. No.50 Donghuan Road,Suz hou215021, Jiangsu Province, P.RChina

## Purpose

Sports park is a kind of modern city park combining sports functions. It integrates sports and fitness venues and ecological environment to create healthy, athletic and recreational park green space. China now has a sports population of 300 million, and there is a great demand for sports. As the carrier of public sports services, sports park provides venues, facilities and services for citizens’ sports. This paper takes the public sports service in Chinese sports parks as the research object, studies the supply problem existing in the public sports service, hopes to promote the efficiency of public services in sports parks, improve the participation of citizens in sports, provide Suggestions for the future development of Chinese sports parks.

## Methods

This paper uses literature, questionnaire, interview and case study. Relevant literature was retrieved through CNKJ, China sports information network and other information platforms. Three sports parks were randomly selected for case study. Field interviews were conducted with park managers. Questionnaires were made and distributed to 300 citizens who participated in exercise in sports parks to investigate the supply subjects, supply financing, service types and supply management of sports parks.

## Results

The service items in the sports park are mainly outdoor fitness track and traditional ball games, with a single form. Lack of indoor stadiums and recreational sports. The capital supply for park construction and development is mainly from the government, the financing channels are single. The park has a low timeliness in the maintenance and protection of the site facilities, and lacks solutions to sudden injury accidents. There is no sports guidance service and no sports guidance staff in the park. Sports activities held in the park are insufficient in number and small in scale. Insufficient publicity for the park, lacking of publicity on the Internet and television.

## Conclusion

The public sports service supply of China’s sports parks is single in content, lacking of venues and facilities, no special guidance personnel and insufficient publicity.

# Abstract: P31: The epidemiology of muscle and tendon injuries during the super rugby tournament (2013 to 2015)

**Published:** 2019-01-01

**Authors:** Hashendra Ramjee, A Jansen van Rensburg, Martin P. Schwellnus, Dina C (Christa) Janse Van Rensburg, Charl Janse Van Rensburg, Esme Jordaan

**Affiliations:** 1 Sport Exercise Medicine and Lifestyle Institute (SEMLI) and Section Sports Medicine, Faculty of Health Sciences, University of Pretoria, Pretoria, South Africa; 2 Emeritus Professor of Sport and Exercise Medicine, Faculty of Health Sciences, University of Cape Town, South Africa; 3 International Olympic Committee (IOC) Research Centre, South Africa; 4 Biostatistics Unit, Medical Research Council, Parow, South Africa; 5 Statistics and Population Studies Department, University of the Western Cape

## Introduction

Rugby union is a high-intensity, contact sport and is therefore associated with significant injuries. Muscle and tendon injuries form a major proportion of these injuries. The aim of this study was to determine the epidemiology of muscle / musculotendinous (M/MT) injuries amongst elite rugby players over a three-year period (2013 – 2015) of the annual Super Rugby Tournament.

## Methods

Elite male South African rugby players (n= 594) that participated in the annual Super Rugby competition, for a period of 3 years. Data collected daily by each team physician during the tournament period, included injuries sustained in training or match-play.

## Results

A total of 51 952 player-hours were monitored over the 3 year period (4 620 match player-hours; 47 333 training player-hours). In total 578 injuries were documented, of which 278 were M/MT injuries (48%) sustained during this period. M/MT injuries accounted for the majority of tissue-type injuries overall, and were more frequent during match-play (IR 51.9/1000 match player-hours (95% CI 45.6 – 59.0) compared to training, over the 3 years of the tournament. The incidence rate of M/MT injuries during match-play for 2013 (68.4/1000 player-hours; 95% CI 55.9 – 82.9) was significantly higher (p<0.05) than in 2014 (35.8/1000 player-hours; 95% CI 26.8 – 46.8) and 2015 (51.2/1000 player-hours; 95% CI 40.8 – 63.5). The most common anatomical site for M/MT injuries is the lower limb, with the mechanism of M/MT injury mostly contact related (tackle). M/MT injury incidence rate is also greater in back player positions when compared to forward positions. Severity of M/MT injuries from a ‘time-loss’ perspective were mostly minimal (2–3 days).

## Conclusion

The results of this study confirm that M/MT injuries form a significant part of the injury burden in South African teams during the annual Super Rugby tournament. Most M/MT injuries occur during match-play.

# Abstract: P32: Race distance, history of chronic disease and allergies are risk factors associated with overuse injuries in trail runners: a SAFER study in 2824 entrants

**Published:** 2019-01-01

**Authors:** Nicola Sewry, Carel Viljoen, Martin Schwellnus, Sonja Swanevelder, Esme Jordaan

**Affiliations:** 1 Sport, Exercise Medicine and Lifestyle Institute (SEMLI), Faculty of Health Sciences, University of Pretoria, South Africa; 2 International Olympic Committee (IOC) Research Centre, South Africa; 3 Sport, Exercise Medicine and Lifestyle Institute (SEMLI) and Department of Physiotherapy, Faculty of Health Sciences, University of Pretoria, South Africa; 4 Emeritus Professor of Sport and Exercise Medicine, Faculty of Health Sciences, University of Cape Town, South Africa; 5 Biostatistics Unit, South African Medical Research Council; 6 Statistics and Population Studies Department, University of the Western Cape, South Africa

## Introduction

Trail runners are an emerging and growing population in the running community, but there are minimal data on the epidemiology and risk factors associated with overuse injuries. The purpose of this study is to determine the risk factors associated with overuse injuries in shorter distance trail runners entering a mass community-based trail running event over a 4-year period.

## Methods

In this cross sectional study, 2824 consenting race entrants from the Two Oceans trail runs (2012–2015, 10km and 22km) completed an online pre-race medical screening questionnaire. Entrants were grouped as having a past (in last 12 months) history of an overuse injury (n=383, with 395 injuries) and a non-injured control group (n=2441) - 8 entrants were excluded, based on missing data. The following risk factors associated with injuries investigated were: demographics, running training/racing history, history of main categories of chronic disease, regular use of medications. A multi-variate model was adjusted for age and gender, and prevalence ratios (PR) and p-values are reported.

## Results

Sex, age categories or training / racing history were not related to overuse injury risk. Independent risk factors that were associated with a higher PR of previous injury were: 1) longer trail run distance (short vs long, PR=0.6; p<0.001), risk factors for CVD (yes vs no, PR=1.6; p=0.0001), history of allergies (yes vs no, PR=1.8; p<0.0001).

## Conclusions

We show that longer race distance, a history of chronic disease and allergies are independent risk factors associated with self-reported overuse injuries in trail runners. These risk factors can influence future injury prevention and management strategies.

# Abstract: P33: The effect of robotic walking and activity-based rehabilitation on secondary complications in individuals with spinal cord injury

**Published:** 2019-01-01

**Authors:** Claire Shackleton, Robert Evans, Sacha West, Wayne Derman, Yumna Albertus

**Affiliations:** 1 Division of Exercise Science and Sports Medicine, Department of Human Biology, Faculty of Health Science, University of Cape Town, Cape Town, Western Cape, South Africa; 2 Department of Sports Management, Cape Peninsula University of Technology, Cape Town, Western Cape, South Africa

## Introduction

Individuals with spinal cord injuries (SCI) are at high risk for a multitude of secondary health complications. Gold standard Activity-based Training (ABT) and novel Robotic Locomotor Training (RLT) show promising results for reducing secondary complications. However, there is a need for increased evidence through large-scale trials. We thus aimed to evaluate the effects of RLT and ABT on secondary complications in individuals with SCI.

## Methods

Sixteen participants aged 19 – 60 years (mean ± SD: 38.4±14.3), with chronic (>1 year) traumatic motor incomplete SCI (C1–C8; ASIA C-D) were randomized into two intervention groups; RLT and ABT groups (control). Each intervention involved 60-minute sessions, 3x per week, over 24-weeks. RLT involved solely walking in the Ekso bionic suit. ABT involved a variety of physical exercises with regular weight bearing in the standing position. Outcome measures included pain and spasticity, bladder and bowel function, and Quality of Life (QoL). Statistical analysis included repeated measures analysis of variance. A p-value of < 0.05 was considered as statistically significant.

## Results

Pain interference with mood (mean difference = −1.56; 95% CI [−2.92 to −0.20]) and day-to-day activities (mean difference = −2.18; 95% CI, [−4.06 to −0.30]) increased across the interventions (p = 0.014). No change in number of pain areas or the intensity of that pain. The shoulder was the most frequent area of pain complaint, followed by the neck and lower back. No changes in total spasticity or bladder and bowel were reported. QoL increased (mean difference −3.68; 95% CI, [−6.21 to −1.15]) across the intervention for both groups (p = 0.003).

## Conclusion

Although the interventions did not show positive effects on pain and spasticity, QoL improved across the 24-weeks for both groups. Thus, the long-term psychological benefit of these rehabilitation interventions is promising.

# Abstract: P34: The association between the mechanism of injury and the visual MRI pathology of ACL injuries

**Published:** 2019-01-01

**Authors:** Org Strauss, Dina C (Christa) Janse Van Rensburg, Mark Velleman, Lizelle Fletcher, Audrey Jansen van Rensburg, Catharina C. Grant

**Affiliations:** 1 Diagnostic Radiology, University of Pretoria, South Africa; 2 Section Sports Medicine, Faculty of Health Sciences, University of Pretoria, Pretoria, South Africa; 3 Little Company of Mary Medical Centre, Pretoria, South Africa; 4 Department of Statistics, Faculty of Natural and Agricultural Sciences, University of Pretoria, Pretoria, South Africa

## Introduction

Anterior cruciate ligament (ACL) injuries are common among athletes and the general public, and may lead to significant absence from activity with an associated financial and social burden. No definitive association has been described between mechanism of injury and pathology, to implement preventative measures to limit these injuries. The aim of this study was to determine whether an association between the mechanism of injury and pathology seen on a magnetic resonance imaging (MRI) scan in ACL injuries, exists.

## Methods

A cross-sectional analytical study of 87 male patients with ACL injury, who had an MRI knee scan within the last two years. Participants were contacted for consent. The mechanism of injury and pathology seen on the MRI scan was noted and categorised into different injury and associated pathology groups.

## Results

MRI scans of ACL injuries indicated the mechanism of a solid foot plant with rotation of the knee has a greater tendency to be associated with medial meniscal injuries (77%), and also a 54% possibility to be associated with lateral meniscal injuries. A solid foot plant with a valgus stress on the knee showed a higher incidence of associated medial collateral ligaments (MCL) injuries (41%) and femoral bone bruising (62 %). These two mechanisms of injury are the most common in ACL injuries and contribute to the clinical significance found. Results was however not statistically significant (p=0.44, chi-square value=20.27, df=45) for any association between pathology and mechanism of injury.

## Conclusion

Some injury mechanisms causing ACL injury were more common than others and also had more associated pathology. The most common mechanism of injury noted is a solid foot plant with either rotation of the knee or valgus stress on the knee. Strengthening tissue structures involved in those movement patterns that cause these mechanisms can possibly limit ACL injuries in athletes and the general public.

# Abstract: P35: Work and family support among participants in the world transplant games

**Published:** 2019-01-01

**Authors:** Prof Johan van Heerden

**Affiliations:** University of KwaZulu-Natal

## Purpose

To conduct an analysis of work and family support systems among participants in the Transplant Games.

## Methods

The design comprised an analytical survey among participants (n=60) taking part in the 19th World Transplant Games, held in Durban, South Africa. Work and family support were measured respectively, using the Modified Work APGAR (Adaptation, Partnership, Growth, Affection, Resolve) and the Smilkstein’s Family System APGAR Questionnaires.

## Results and Conclusion

There was a greater prevalence of liver and kidney transplants than any other organ. APGAR scores showed high functionality. Females were more functional with regards to work and family support (p>0.05). Kidney transplant recipients had greater functionality in the work place; however, liver transplant recipients had a greater functionality with regards to family support (p<0.05). Participants waiting more than two years for a transplant showed greater functionality with regards to work and family support (p>0.05).

In conclusion this study provides additional information with regards to work and family support systems and their functionality among transplant recipients who are physically active.

# Abstract: P36: Prevalence and type of injuries in South African trail runners

**Published:** 2019-01-01

**Authors:** Harry Van Wyk, Steyn Swart, Susan H Bassett

**Affiliations:** Department of Sport, Recreation and Exercise Science, University of the Western Cape, Cape Town, South Africa

## Introduction

Trail running is becoming an ever more popular sport worldwide with the trail running population having more than doubled since 2006. More and more people are moving from the monotonous and repetitive running nature of road running to the trails. With the popularity of trail running increasing, so are the number of injuries being reported. Injuries range from acute to chronic and include muscular, skeletal, ligamentous and tendinous. The amount of time an athlete cannot train or compete due to injury depends on its severity. This study aims to investigate the prevalence and type of injuries of trail runners in South African trail runners aged between 19–40 years.

## Methods

This study will employ a descriptive, survey research design involving the use of a questionnaire sent to various trail running groups or clubs, which will be used to collect specific data from the runners. The sample will include male and female trail runners from the ages of 19–40 years from both recreational to elite level, who have at least 2 years of running experience. An information sheet, explaining the study and what the participant will be asked to do, will be sent via email to various trail running groups and clubs across the country to distribute to their members. Participation will be purely voluntary and a link will be provided, via the running club, to the Google Form questionnaire. By completing the questionnaire, consent will be implied. The sample size is dependent on the amount of respondents. Descriptive statistics will be utilized to analyse the data. In doing so, the type of injury and prevalence thereof will be established.

## Results

This study data has not be collected yet, but we aim to do this by September this year, so the study will be complete in time for presentation at the conference in October.
